# An Ocular Surface‐Targeting Nucleic Acid Hydrogel for Efficient Dry Eye Disease Treatment

**DOI:** 10.1002/EXP.20240461

**Published:** 2026-07-15

**Authors:** Yuhe Liu, Zimeng Zhai, Yangyang Huang, Fujun Wang, Zifei Wang, Lijuan Zhu, Ka Jiang, Xujiao Zhou, Xingtao Zhou, Ying Jie, Xiuming Jin, Chuan Zhang, Jiaxu Hong

**Affiliations:** ^1^ School of Chemistry and Chemical Engineering State Key Laboratory of Synergistic Chem‐Bio Synthesis Shanghai Key Laboratory for Molecular Engineering of Chiral Drugs Shanghai Jiao Tong University Shanghai P. R. China; ^2^ Department of Ophthalmology and Vision Science State Key Laboratory of Brain Function and Disorders, MOE Frontiers Center for Brain Science Shanghai Eye, Ear, Nose and Throat Hospital; NHC Key laboratory of Myopia and Related Eye Diseases; Shanghai Key Laboratory of Gene Editing and Cell Therapy for Rare Diseases Fudan University Shanghai P. R. China; ^3^ School of Life Sciences Fudan University Shanghai P. R. China; ^4^ Beijing Institute of Ophthalmology Beijing Tongren Eye Center Beijing Ophthalmology & Visual Sciences Key Laboratory Beijing Tongren Hospital Capital Medical University Beijing China; ^5^ Eye Center, The Second Affiliated Hospital of Zhejiang University, School of Medicine Hangzhou China; ^6^ Department of Ophthalmology Children's Hospital of Fudan University Shanghai P. R. China; ^7^ Department of Ophthalmology Jinjiang Second Hospital Quanzhou Fujian P. R. China

**Keywords:** dry eye disease, nucleic acid hydrogel, ocular surface‐targeting, oligonucleotide drug, siRNA delivery

## Abstract

Dry eye disease (DED) is a prevalent disorder affecting millions worldwide, fueled by both the aging population and extensive use of electronic devices. Yet, current treatments for moderate‐to‐severe DED fail to fully meet the clinical needs due to the lack of effective therapeutics and delivery systems for topical application. Based on the concept of “Barrier to Target,” an siRNA‐embedded nucleic acid hydrogel modified with a mucin‐1 (MUC1) aptamer is developed to actively target the ocular surface for DED treatment. Upon distillation, the adhesive feature and aptamer‐mucin recognition of the hydrogel enhance its retention on the ocular surface. Over time, tear dilution and the shear force of blinking lead the hydrogel to degrade into nanosized gel particles, facilitating drug uptake by corneal cells. Subsequently, the embedded siRNA targeting the nuclear factor of kappa‐B inhibitor, zeta (NFKBIZ) gene can effectively silence the target gene expression, initiating a cascade of inflammation‐related gene suppression. This comprehensive gene regulation effectively reshapes the inflammatory corneal environment to a normal state and alleviates the severity of DED in a mouse model. With the sequence‐dependent nature of siRNA drugs, our targeting hydrogel may serve as a general platform for treating various ocular surface diseases.

## Graphical abstract

A self‐assembled nucleic acid hydrogel modified with mucin‐1 (MUC1) aptamer is developed, which can actively target the mucin layer on the ocular surface and significantly extend its retention time after distillation. Inside the hydrogel, therapeutic siRNA is embedded and can effectively silence the inflammation gene expression, subsequently restoring a balanced immune microenvironment of the ocular surface and achieving an ideal therapeutic outcome in dry eye disease treatment.

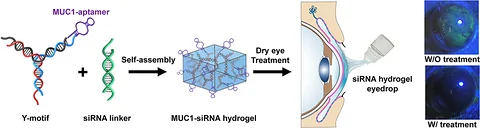

## 1 Introduction

Dry eye disease (DED) is a widespread ocular surface condition affecting 5%–50% of the global population, often resulting in severe ocular discomfort and visual impairment [1]. The prevalence of DED is on the rise, with inflammation playing a key role in its multifactorial progression, perpetuating a vicious cycle of deterioration [2]. Despite advances in its underlying mechanism, clinical treatment remains limited. Current medicines primarily include artificial tears for mild DED and immunosuppressants like glucocorticoids or cyclosporine (CsA) for moderate‐to‐severe manifestations [3, 4]. However, conventional treatments, predominantly administered via eye drops, still face challenges in practice. For instance, the presence of a tight corneal barrier usually leads to short residence time and low bioavailability (5%–10%) of topically applied drugs, which necessitates high drug dosage and results in rebound symptoms upon discontinuation [5]. Furthermore, long‐term use of anti‐inflammatory agents carries the risk of low adherence of patients and adverse events such as ocular pain, hyperemia, and increased intraocular pressure [6]. Consequently, there is an urgent need to devise novel strategies for effective DED treatment.

Among various emerging DED therapeutics, clinical trials of Tivanisiran, an siRNA used for DED treatment, have provided evidence that nucleic acids may serve as potent therapeutics in ophthalmology [7]. However, the development of Tivanisiran as a commercial drug has encountered delays and setbacks. Its latest clinical outcomes still fall short of satisfactory therapeutic efficacy, prompting a new clinical trial by only recruiting DED patients with Sjögren syndrome for further investigation [8]. Unlike small molecular drugs, siRNA molecules are known for their relatively large molecular weight and highly negatively charged feature, presenting greater challenges in overcoming the ocular surface barrier and efficient delivery of siRNA to corneal tissue. As an early exploration, Tivanisiran, without the use of any carriers or modifications, relies on high drug concentration to passively diffuse into the corneal surface, which may partially explain the relatively low efficacy observed in clinical trials [9]. While viral vectors, lipid nanoparticles, synthetic polymers, and inorganic nanoparticles have been explored for siRNA delivery in the past two decades [10, 11, 12, 13, 14, 15], the cornea and conjunctiva, as the largest ocular surface barrier, pose a formidable challenge for topical drug delivery, hindering the drug penetration into ocular tissues and contributing to most treatment failures [16]. Furthermore, the sensitivity of the ocular surface requires careful consideration when selecting carriers to avoid potential irritation, limiting the types of carriers suitable for topical applications [17]. Besides gene regulatory agents, nucleic acid itself can serve as carrier materials when assembled into specific nanostructures and loaded with functional nucleic acid drugs inside. A large variety of self‐assembled DNA nanostructures, including nucleic acid frameworks, DNA origami, hydrogels, and spherical nucleic acids, have been developed and utilized for delivering various therapeutic nucleic acids across a broad spectrum of diseases [18, 19, 20, 21]. Recent studies highlight the aptamers as promising affinity ligands to enhance ocular surface retention and delivery efficiency. For instance, mucin‐binding aptamers [e.g., specifically targeting mucin‐1 (MUC1)] [22] and stimuli‐responsive aptamer systems [23] have demonstrated the improved delivery of therapeutic agents. These approaches, however, often lack well‐controlled precise drug loading or sustained release kinetics, impeding their future clinical translation.

**SCHEME 1:**
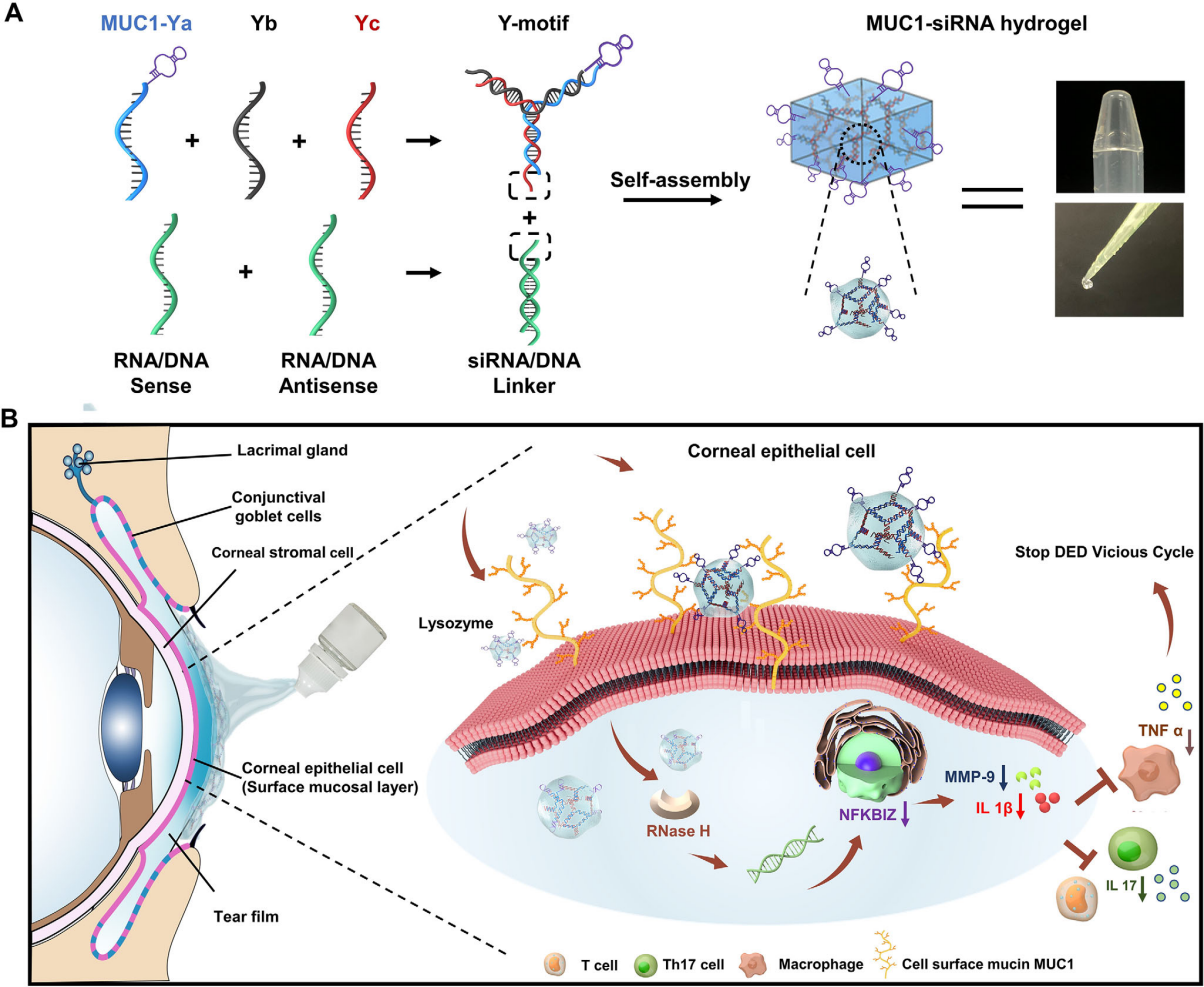
Schematic illustration of MUC1 aptamer‐modified and siRNA‐bearing nucleic acid hydrogel (MUC1‐siRNA hydrogel) for DED treatment. (A) The design and self‐assembly of the MUC1‐siRNA hydrogel; dashed boxes indicate the complementary sticky ends in the Y‐motif and siRNA or DNA linker (DNA linkers are employed to partially replace the siRNA linker to adjust the siRNA concentration in the hydrogel used for DED treatment in a mouse model). (B) The targeted delivery of MUC1‐siRNA hydrogel and its therapeutic mechanism to alleviate the DED inflammation via target gene silencing.

To address these limitations, we developed a self‐assembled nucleic acid hydrogel bearing therapeutic siRNAs for topical delivery. Nucleic acid hydrogels have demonstrated unique advantages in fields such as cancer vaccines and tissue repair through long‐lasting sustained release, precise local delivery, multi‐functional integration, and biocompatibility design, making them a promising delivery system complementary to conventional liposome‐ and LNP‐based carriers or viral vectors [24]. In our design, the nucleic acid hydrogel is simply synthesized by programmable self‐assembly using a Y‐shaped DNA motif (Y‐motif) and an siRNA linker via sticky‐end association (see Graphical abstract). Given that the cornea comprises layers of epithelial cells covered with membrane‐associated mucin proteins, including MUC1, MUC4, and MUC16 [25, 26], we engineered an MUC1 aptamer into the Y‐motif backbone to leverage active mucin targeting on the ocular surface [27, 28, 29]. Unlike conventional aptamer conjugates, our design integrates the aptamer within a programmable hydrogel architecture, enabling synergistic sustained siRNA release and enhanced cellular uptake. Moreover, considering the pathogenesis of DED characterized by persistent inflammation [30, 31], the siRNA sequence in the present study was designed to target the nuclear factor of kappa‐B inhibitor, zeta (NFKBIZ) gene, which encodes the vital transcriptional coactivator IκBζ that mediates a series of downstream pro‐inflammatory cytokine expression, such as IL‐6, IL‐1β, tumor necrosis factor (TNF)‐α, and IL‐17 [32]. Equipped with aptamer and regulatory siRNA, our nucleic acid hydrogel exhibits remarkable targeting capability to the ocular surface and efficient knockdown of NFKBIZ gene expression, thereby remodeling the inflammatory cornea into a normal state in a DED mouse model. Compared to CsA eye drops and existing aptamer‐based delivery systems [22, 23, 33], our hydrogel uniquely combines MUC1‐specific targeting, sustained siRNA release, and target gene regulation into a single platform, which can effectively manage the DED‐associated inflammation and significantly reduce the DED severity in a mouse model. Based on the strategy of “Barrier to Target” and taking the advantages of the sequence‐dependent feature of siRNA drugs, our targeting nucleic acid hydrogel not only offers an alternative to relieve moderate‐to‐severe DED but also holds promise for expanding its applications across a wide spectrum of ocular surface diseases by changing the siRNA sequences (See Scheme 1).

## 2 Results

### 2.1 Syntheses and Characterizations of MUC1‐siRNA Hydrogel

**FIGURE 1:**
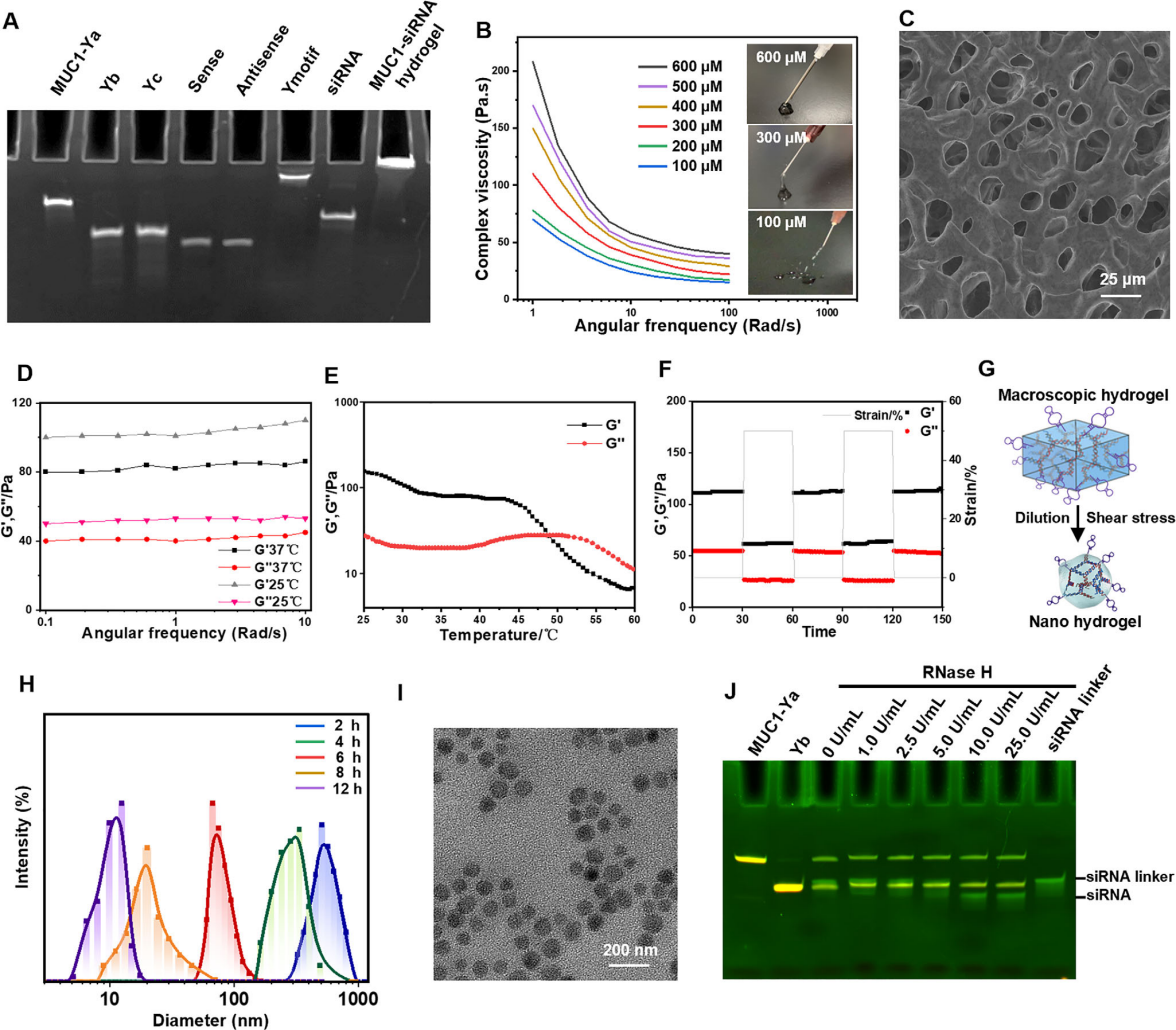
Characterizations of MUC1‐siRNA hydrogel and the enzyme‐mediated siRNA release. (A) 20% native PAGE gel electrophoresis of the prepared MUC1‐siRNA hydrogel. (B) Viscosity vs. frequency scanning in rheological tests for MUC1‐siRNA hydrogel with different gelation concentrations (varied linker concentrations). Inset: Injectable feature of 600, 300, and 100 µM MUC1‐siRNA hydrogel. (C) The morphology of the 300 µM MUC1‐siRNA hydrogel determined by SEM. (D–F) Rheological properties of 300 µM MUC1‐siRNA hydrogel. (D) Storage modulus and loss modulus (G′ and G″) from temperature sweep, (E) G′ and G″ from angular frequency sweep, and (F) G′ and G″ from stress‐time cycle. (G) Schematic diagram of the degradation of a hydrogel. (H) The hydrodynamic diameter of degraded MUC1‐siRNA hydrogels after 2‐, 4‐, 6‐, 8‐, and 12‐h stirring and incubation, which were analyzed by dynamic light scattering (DLS). (I) The transmission electron microscopy (TEM) image of MUC1‐siRNA hydrogel diluted (100‐fold dilution) by a tear‐mimic, lysozyme‐containing PBS buffer (2.4 mg·mL^−1^) with slow stirring at 37°C for 6 h. (J) 15% denaturing gel analysis of MUC1‐siRNA nanogel after incubation with different concentrations of RNase H for 1.0 h at 37°C. Scale bar: as shown in images.

As illustrated in the graphical abstract, the ocular surface‐targeting hydrogel for DED treatment is simply synthesized through the programmable self‐assembly using a typical Y‐shape DNA motif and a linear siRNA crosslinker [34]. To enable its active targeting capability, we specifically introduce an MUC1‐aptamer segment at one end of component strand A (MUC1‐Ya). By mixing MUC1‐**Ya** with component strand B (**Yb**) and strand C (**Yc**) at a 1:1:1 ratio in 1× tris‐acetate‐ethylenediaminetetraacetic acid (EDTA)‐Mg^2+^ (TAE/Mg^2+^) buffer and annealing from 90°C to room temperature, the Y‐motif building blocks could be obtained as verified by 20% native polyacrylamide gel electrophoresis (PAGE; Figure 1A). Meanwhile, siRNAs with sequences targeting either the human NFKBIZ gene (anti‐h‐NFKBIZ siRNA, or siNFKBIZ‐H) or the mouse NFKBIZ gene (anti‐m‐NFKBIZ siRNA, or siNFKBIZ‐M) were designed into crosslinkers by introducing a 12nt overhang at each end with a sequence complementary to the sticky ends on the Y‐motif. Once these two building blocks were ready, the Y‐motif and siRNA crosslinker were mixed at a 1:1.5 ratio at room temperature, resulting in the formation of a MUC1 aptamer‐equipped hydrogel (**MUC1‐siRNA hydrogel**) through sticky end hybridization. Specifically, to tune the siRNA concentration in the hydrogel used for DED treatment in a mouse model, we partially replaced the siRNA crosslinkers with DNA crosslinkers of the same sequence during the hydrogel self‐assembly. Owing to its large network structure, the hydrogel was stuck in the well during the gel electrophoresis (Figure 1A). As a comparison, component strand A without the aptamer segment (Ya) was also employed for the hydrogel assembly, resulting in the formation of a control hydrogel (**siRNA hydrogel**). Meanwhile, an MUC1 aptamer‐containing hydrogel without gene regulatory capability was synthesized using an siRNA linker with a scramble sequence (**MUC1‐Scr hydrogel**; Figure S1 and see all detailed sequences in Table S1). In general, the hydrogel gelation process was rapid and could be completed within 2 min. Their rheological features are highly dependent on the concentrations of building blocks used during the assembly. To screen a hydrogel with suitable fluidity and transparency for topical application, a series of MUC1‐siRNA hydrogels assembled at different concentrations (ranging from 100 to 600 µM in terms of linkers) were constructed, following with characterizations. As shown in Figure 1B, the hydrogel assembled at 600 µM exhibits as a solid substance with almost no injectability, while the hydrogel assembled at 100 µM shows low viscosity and is easily swept away. In contrast, the hydrogel with medium concentration (300 µM) possesses moderate viscosity and ideal injectable features when subjected to shear forces, which is highly aligned with the rheological tests (Figure 1B). Notably, scanning electron microscopy (SEM) imaging illustrates a typical porous network structure of MUC1‐siRNA hydrogel (Figure 1C), again confirming the formation of hydrogel through nucleic acid self‐assembly. With the moderate hydrogel (300 µM), we further investigated its rheological properties on a rotational rheometer. Frequency sweep tests (at a fixed strain of 1%) implemented between 0.1 and 10 rad·s^−1^ were performed at both 25°C and 37°C, respectively. As shown in Figure 1D, the storage modulus (G′) exhibits a higher value at lower temperatures and is apparently higher than the corresponding G″ in both cases, confirming the real hydrogel feature in the physiological environments. Moreover, temperature‐ramp rheological tests conducted from 25°C to 60°C (at a frequency of 1 Hz and a strain of 1%) revealed a modulus (storage modulus G′ and loss modulus G″) with temperature‐dependent behavior for the obtained moderate MUC1‐siRNA hydrogel (Figure 1E). Specifically, with the increase of temperature from 25°C to 60°C, G″ gradually decreased and intersected with the G″ curve at 48°C, revealing the transition of hydrogel from gel to sol state because of the nucleic acid disassembly. In addition, stress‐time scanning revealed that the G′ and G″ could be recovered when different stress was applied with multiple cycles (Figure 1F). With tunable rheological properties, the prepared hydrogel with optimized viscosity (gelation with 300 µM linker) is employed as a candidate for ocular surface administration and targeted therapeutic siRNA delivery for the following investigations.

As a macroscopic hydrogel, it is difficult to directly deliver the component siRNA therapeutics if the hydrogel remains intact. A previous study demonstrated that the self‐assembled nucleic acid hydrogel is a biodegradable drug delivery system, where dilution and enzymatic digestion will induce the gradual degradation of the nucleic acid hydrogel into nanosized gel particles [24, 35]. When topically applied to the ocular surface, the continuously secreted tear will greatly dilute the hydrogel. Meanwhile, the shear stress of blinking and tear‐containing enzymes may also facilitate its degradation (Figure 1G). To verify our hypothesis, we incubated the MUC1‐siRNA hydrogel with PBS buffer containing 2.4 mg·mL^−1^ lysozyme (a major enzyme in tear fluid) under continuous stirring (200 rad·min^−1^) to simulate the ocular surface physiological environment. After incubation for varied times, samples were subsequently analyzed with multiple techniques. First, agarose gel electrophoresis (1% w/w) showed that the macroscopic gel gradually degraded into smaller assemblies with the extended incubation and stirring time, as verified by bands with increased mobility in the gel (Figure S3A). In the meantime, PAGE (15% w/v) under native conditions showed that all incubated samples were trapped in the loading well even after incubating for 12 h, indicating MUC1‐siRNA hydrogel remained as assembled architectures rather than individual motifs (Figure S3B). Transmission electron microscopy (TEM) imaging also showed that MUC1‐siRNA hydrogel indeed degraded into smaller particles during the mimic degradation process. As incubation time goes on, particles with gradually decreased sizes (ranging from 1 µm to ∼20 nm) could be observed in the TEM images and dynamic light scattering (DLS) measurements (Figure S3C–F and Figure 1H). The obtained nanogel particles were also confirmed by TEM and atomic force microscopy (AFM; Figure 1I and Figure S2). Furthermore, to confirm the enzyme‐mediated siRNA release from the MUC1‐siRNA hydrogel after cellular uptake, we incubated the hydrogel with different concentrations of RNase H at 37°C for 1 h and followed with gel analysis using 15% denaturing PAGE gel. In the presence of RNase H, MUC1‐siRNA hydrogel was able to release siRNA in a concentration‐dependent manner (Figure 1J), which is in line with our previous findings [24]. All results above demonstrated that our MUC1‐siRNA hydrogel possesses suitable mechanical and rheological properties, which could be degraded into nanoparticles under physiological conditions. With adhesive retention at the macroscopic state and penetration capability of nanogel morphology, our hydrogel may serve as an ideal siRNA delivery system for topical application.

### 2.2 In vitro Targeting Capability of MUC1‐siRNA Hydrogel

**FIGURE 2:**
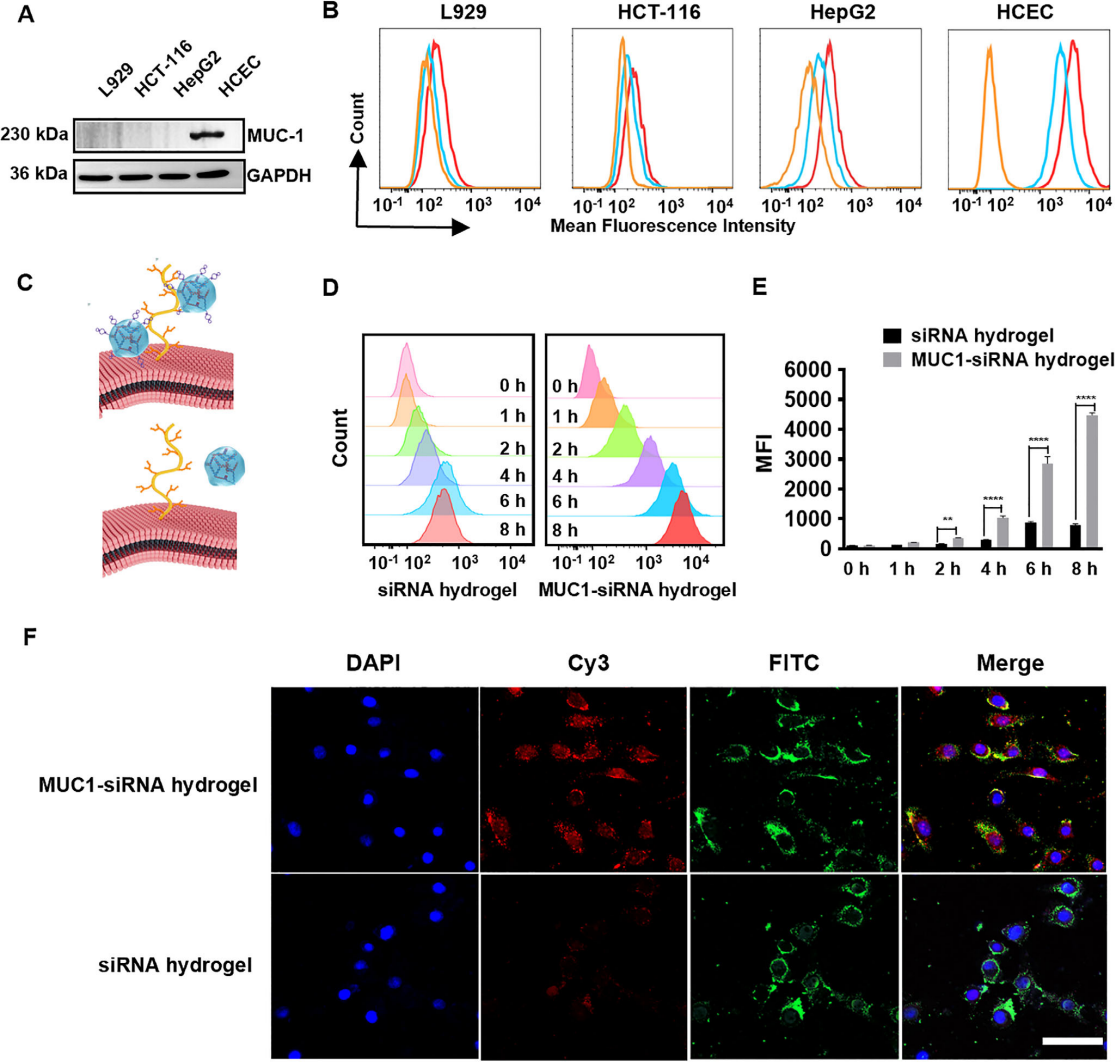
The targeting capability and cellular uptake behaviors of MUC1‐siRNA hydrogel. (A) MUC1 expression in L929, HCT‐116, HepG2, and HCEC cells verified by Western blot analysis. (B) Flow cytometry analyses of L929, HCT‐116, HepG2, and HCEC cells incubated with MUC1‐siRNA hydrogel (300 µM gelation) at different concentrations (siRNA‐antisense linker was labeled with Cy3; red curve: 2 µM siRNA (150‐fold dilution), blue curve: 1 µM siRNA (300‐fold dilution); yellow curve: mock). (C) Schematic diagram of the uptake of hydrogel. (D) Flow cytometry analysis of HCEC cells treated with MUC1‐siRNA hydrogel (right) and siRNA hydrogel (left) for different incubation times. (E) The mean fluorescence intensities of HCEC cells treated with MUC1‐siRNA hydrogel and the control sample for different incubation times (with equivalent Cy3 concentrations: 1 µM). Data were presented as mean ± SD; *n* = 3. Multiple *t*‐tests statistical significance: **p < 0.01, ****p < 0.0001. (F) In vitro HCEC cellular uptake behaviors of MUC1‐siRNA hydrogel and pristine siRNA hydrogel (siRNA linker was labeled with Cy3 at an equivalent concentration of 1 µM) determined by immunofluorescence (membrane‐associated MUC1 was visualized by immunostaining using FITC‐labeled MUC1 antibody) and CLSM imaging after 6 h incubation. Scale bars: 20 µm.

After synthesis, we first investigated the targeting capability of MUC1‐siRNA hydrogel. Prior to the cellular uptake study, expression of MUC1 protein on human corneal epithelial cells (HCEC) was confirmed by Western blot assay, where MUC1‐negative cell lines [L929, human colon cancer cells (HCT‐116), and human hepatoma cells (HepG2 cells)] from other tissues were used as controls. As shown in Figure 2A, L929, HCT‐116, and HepG2 cells show almost no expression of MUC1. In contrast, HCEC cells exhibit a relatively high MUC1 expression, which is consistent with the literatures [36, 37, 38]. The high level of MUC1 expression on HCEC could also be directly visualized by immunofluorescence staining and imaged by confocal laser scanning microscopy (CLSM; Figure 2F). Upon incubating with Cy3‐labeled MUC1‐siRNA hydrogel (Cy3‐labeled siRNA), the MUC1‐positive HCEC cells show strong fluorescent signals in flow cytometry analysis. In contrast, those MUC1‐negative cells displayed a lower cell‐uptake activity when incubated with MUC1‐siRNA hydrogel owing to the lack of MUC1 expression on the surface (Figure 2B). In a systematic cellular uptake study, HCECs were incubated with Cy3‐labeled MUC1‐siRNA hydrogel for different times (1, 2, 4, 6, and 8 h), and pristine hydrogel without an aptamer was used as a control at the same time. As analyzed by flow cytometry, mean fluorescence intensities of the cells treated with MUC1‐siRNA hydrogel gradually increased along with the incubation time, which is significantly higher than that of the hydrogel without aptamer (6.1‐fold at 8 h post‐incubation; Figure 2D,E). In the meantime, the uptake of MUC1 hydrogel by HCEC cells over time was also visualized by CLSM imaging (Figure S4A). Consistent with the flow cytometry, the intracellular fluorescent signal in HCECs gradually enhanced with the increase of incubation time, reaching the maximum after 6–8 h of incubation. As a comparison, minimal red fluorescence can be observed for pristine hydrogel without the aptamer modification, illustrating that the MUC1 aptamer substantially facilitates the internalization of hydrogel particles through receptor‐mediated endocytosis (Figure 2C,F). Note that no obvious fluorescence can be noticed in those three MUC1‐negative cells after 8‐h incubation with Cy3‐labeled MUC1‐siRNA hydrogel (Figures S4B and S5), which further confirms the active targeting capability of the siRNA hydrogel after decoration with the MUC1 aptamer. Besides the corneal epithelial cells, we also examined the interaction between our hydrogel and conjunctival cells. As analyzed by flow cytometry, mean fluorescence intensities of the conjunctival cells (HconEpic cells) treated with MUC1‐siRNA hydrogel were also much higher than those without aptamer modification over the incubation time (Figures S6A,B). Consistent with the flow cytometry results, CLSM imaging revealed the enhanced cellular uptake of MUC1‐siRNA hydrogel in HconEpic cells (Figure S6C). These results indicate that the targeting aptamer can effectively improve the cellular uptake of the nucleic acid hydrogel into both corneal and conjunctival cells, enabling it to serve as a novel carrier for targeted siRNA delivery and gene silencing.

### 2.3 In vitro NFKBIZ Gene Silencing by MUC1‐siRNA Hydrogel and Its Anti‐Inflammatory Effects

**FIGURE 3:**
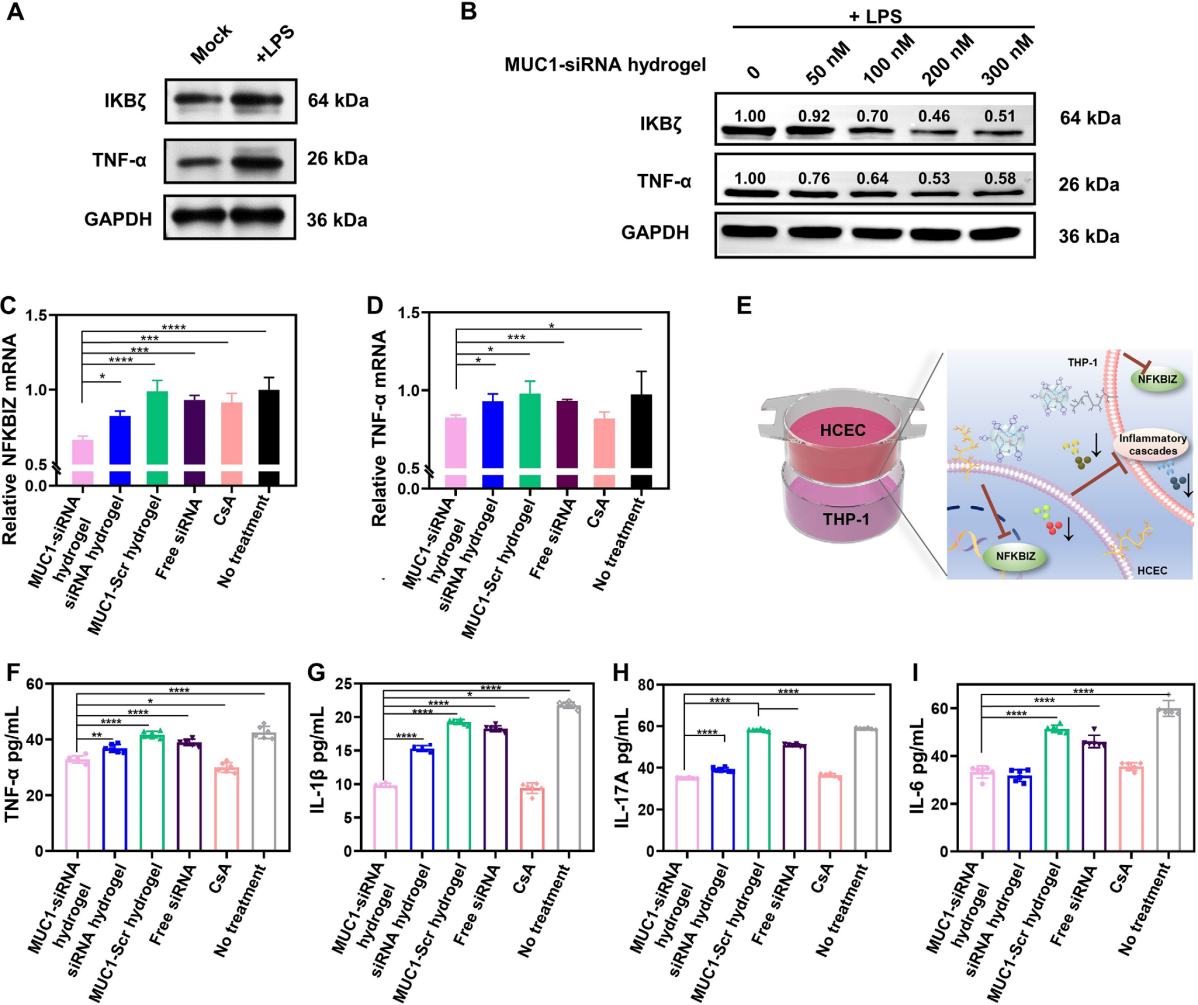
NFKBIZ gene knockdown and anti‐inflammatory effects induced by MUC1‐siRNA hydrogel in vitro. (A) Western blot analysis of IKBζ and TNF‐α expression in HCECs before and after LPS stimulation. (B) Western blot analysis of IKBζ and TNF‐α expression in HCEC cells after treatment with different concentrations of MUC1‐siRNA hydrogel post‐LPS stimulation. (C, D) mRNA levels of NFKBIZ and TNF‐α determined by quantitative reverse transcription‐polymerase chain reaction (qRT‐PCR) analysis, respectively. Cells were treated with MUC1‐siRNA hydrogel with 200 nM siRNA (1500‐fold dilution). Data were presented as mean ± SD; *n* = 3. Statistical significance: *p < 0.05, ***p < 0.001, ****p < 0.0001. (E) Schematic diagram of the proposed mechanism of the inhibition of intracellular signaling pathways and molecular targets. (F–I) ELISA of the secretion of TNF‐α, IL‐1β, IL‐17A, and IL‐6 from THP‐1 and HCEC co‐culture medium. Cells were treated with MUC1‐siRNA hydrogel and control samples with 200 nM siRNA after dilution. Data were presented as mean ± SD; *n* = 3. Statistical significance: *p < 0.05, **p < 0.01, and ****p < 0.0001.

Based on efficient uptake of MUC1‐siRNA hydrogel by HCEC cells, its target gene silencing capability was evaluated at both protein and mRNA levels in vitro. As shown in Figure 3A, upon stimulation with lipopolysaccharide (LPS; 2 µg·mL^−1^), an inflammatory response can be noticed as evidenced by the upregulated expression of TNF‐α and the target co‐transcriptional activator IKBζ. Following the treatment with different concentrations of MUC1‐siRNA hydrogel, the downregulation of TNF‐α and IKBζ in HCEC cells was observed in a dose‐dependent manner, in which the knockdown efficiency could reach up to ∼60% when the siRNA concentration exceeded 200 nM (Figure 3B). In contrast, when treating the cell using a hydrogel containing siRNA with a scramble sequence (MUC1‐Scr hydrogel), no obvious change in IKBζ expression could be found, confirming that the target gene silencing is based on the sequence‐dependent RNAi process. Moreover, IKBζ expression in cells treated with free siRNA and pristine siRNA hydrogel slightly decreased, probably owing to their insufficient cellular uptake by HCEC cells (Figure S7A). A similar pattern in mRNA expression of NFKBIZ was observed after incubating the cells with MUC1‐siRNA hydrogel (Figure 3C). Quantitative reverse transcription‐polymerase chain reaction (qRT‐PCR) results demonstrated that the MUC1‐siRNA hydrogel–treated group had the lowest expression levels of NFKBIZ, among all groups. Besides the target gene, the local silencing of NFKBIZ also triggered a cascade of inhibitory secretion of proinflammatory cytokines, including TNF‐α, IL‐6, IL‐8, and matrix metalloproteinases, such as MMP‐9, a crucial protein to cause cornea damage (Figure 3B,D and Figure S7B–E) [39]. To better mimic the ocular surface microenvironment interactions between immune cells and epithelial cells in vitro, THP‐1 cells (a human macrophage cell line) were co‐cultured with the HCECs in a transwell chamber and then treated with MUC1‐siRNA hydrogel and control samples following the literature protocol (Figure 3E) [40]. With LPS stimulation to the co‐culture system, THP‐1 and HCEC activations and their responses were analyzed by measuring the concentrations of proinflammatory cytokines in the culture media (TNF‐α, IL‐1β, IL‐17A, and IL‐6). After co‐cultivation, enzyme‐linked immunosorbent assay (ELISA) analyses showed that the levels of TNF‐α, IL‐1β, IL‐17A, and IL‐6 cytokines were all downregulated in the MUC1‐siRNA hydrogel–treated group, which was comparable to the effect of commercial CsA eye drops (Figure 3F–I). In contrast, MUC1‐Scr hydrogel exhibits almost no effect on reducing the cytokine secretions. Meanwhile, the non‐targeted hydrogel and free siRNA demonstrated a trivial reduction of cytokines, which may be ascribed to their relatively low uptake efficiency. Similarly, the effective knockdown of MMP‐9 expression by MUC1‐siRNA hydrogel was also confirmed by Western blot analysis, which was significantly superior to other siRNA formulations (Figure S8). Collectively, the efficient silencing of NFKBIZ induced by our MUC1‐siRNA hydrogel greatly alleviates the innate immune response and reduces the proinflammatory cytokine secretions, laying the foundation for its combinatorial DED treatment applied in vivo.

### 2.4 Retention Behavior of MUC1‐siRNA Hydrogel at the Mouse Ocular Surface

**FIGURE 4:**
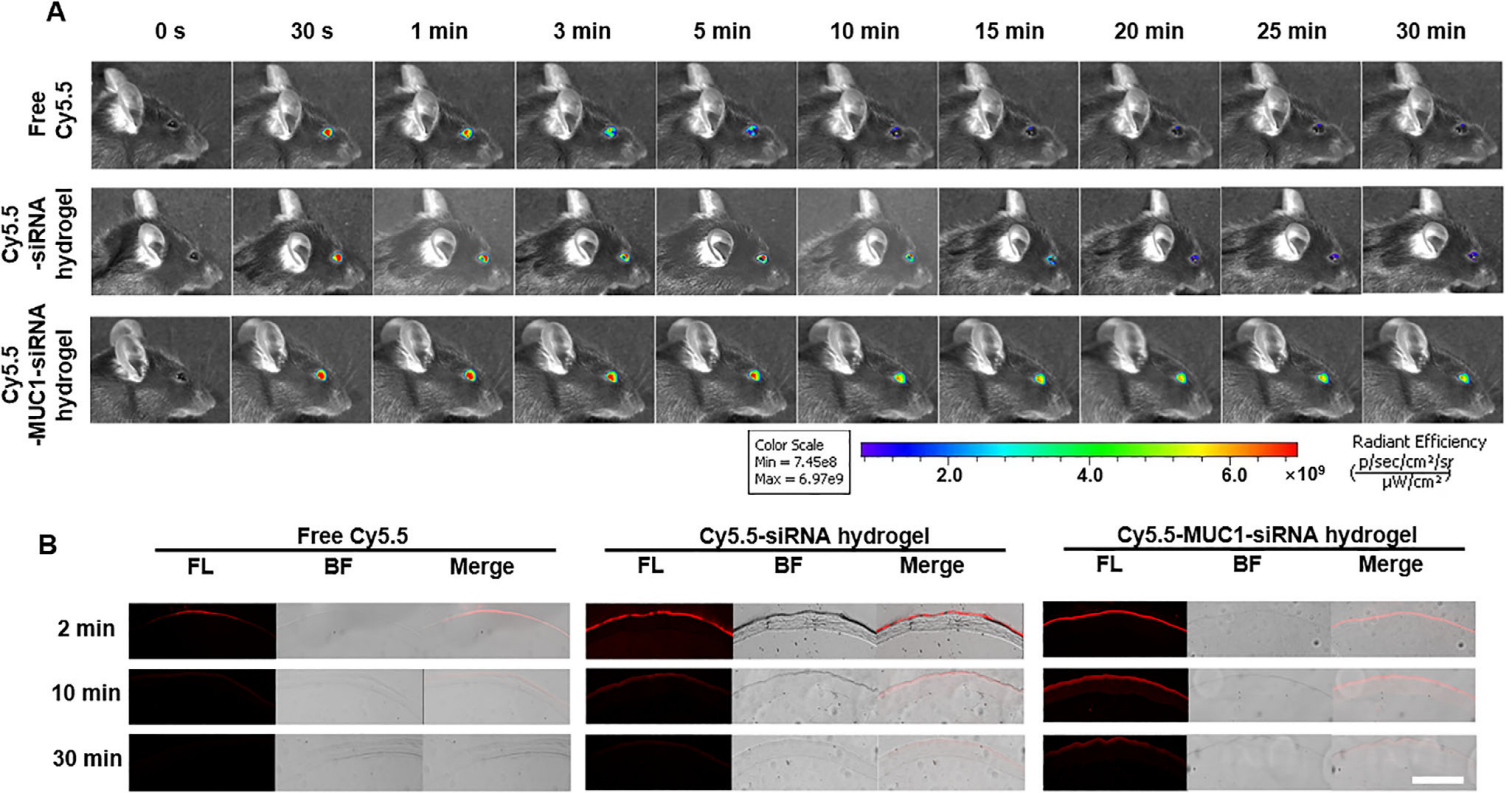
The precorneal retention of MUC1‐siRNA hydrogel on the ocular surface. (A) Real‐time in vivo fluorescence imaging of free Cy5.5, Cy5.5‐labeled MUC1‐siRNA hydrogel (Cy5.5‐MUC1‐siRNA hydrogel) and Cy5.5‐labeled siRNA hydrogel (Cy5.5‐siRNA hydrogel, with an equivalent Cy5.5 concentration: 0.3 mm) on the eyes of anesthetized mice at different time points (0, 0.5, 1, 3, 5, 10, 15, 20, 25, and 30 min). (B) Fluorescence images of frozen cross sections of corneas after administration of Cy5.5‐MUC1‐siRNA hydrogel, Cy5.5‐siRNA hydrogel, and free Cy5.5 to the eyes of mice under conscious states for 2, 10, and 30 min. The scale bar is 50 µm (equivalent Cy5.5 concentration: 0.3 mM).

To verify the active targeting and adhesive capability of the MUC1‐siRNA hydrogel in vivo, the real‐time in vivo fluorescence imaging was applied to intuitively track the retention time of the targeting hydrogel on the ocular surface. After administering Cy5.5‐labeled MUC1‐siRNA hydrogel, siRNA hydrogel, and free Cy5.5 to the eyes of anesthetized mice, fluorescence images were taken at different timepoints (Figure 4A). In the first 30 s, almost identical fluorescence intensities could be noted in all groups owing to the same starting Cy5.5 concentrations. As time passed by, the fluorescence intensity rapidly decreased for both Cy5.5 and non‐targeting hydrogel groups, although the latter one maintained fluorescence for a relatively longer time. In comparison, the fluorescence intensity of the MUC1‐siRNA hydrogel group declined slowly and remained strong even at 30 min post‐administration. To better simulate real‐world usage, the precorneal retention test was conducted on healthy mice without anesthesia. After topically applying different samples, the eyeballs were harvested at different time points and frozen‐sectioned for subsequent analyses. As shown in Figure 4B and Figure S10A, 2 min after distillation, both the MUC1‐siRNA hydrogel and the pristine hydrogel‐treated group showed strong red fluorescence on the corneal surface, whereas the free Cy5.5‐treated group exhibited faint signals, which might be ascribed to the adhesive feature of the hydrogel to prevent the rapid clearance through blinking. At 10 min post‐administration, strong red fluorescence remained in the MUC1‐siRNA hydrogel group. In contrast, fluorescent signals were greatly diminished in the non‐targeting hydrogel group. As time increased, the non‐targeting hydrogel became almost invisible at 30 min, while a clear fluorescent layer could still be noticed over 30 min in the MUC1‐siRNA hydrogel group, demonstrating the significantly prolonged retention of the targeting hydrogel on the ocular surface. The above results verified that our aptamer‐equipped hydrogel could actively target the cornea and promote the drug retention at the ocular surface in vivo, which enables the hydrogel as a promising delivery system for topical application.

### 2.5 In Vivo Therapeutic Effects of MUC1‐siRNA Hydrogel on DED Mouse Model

**FIGURE 5:**
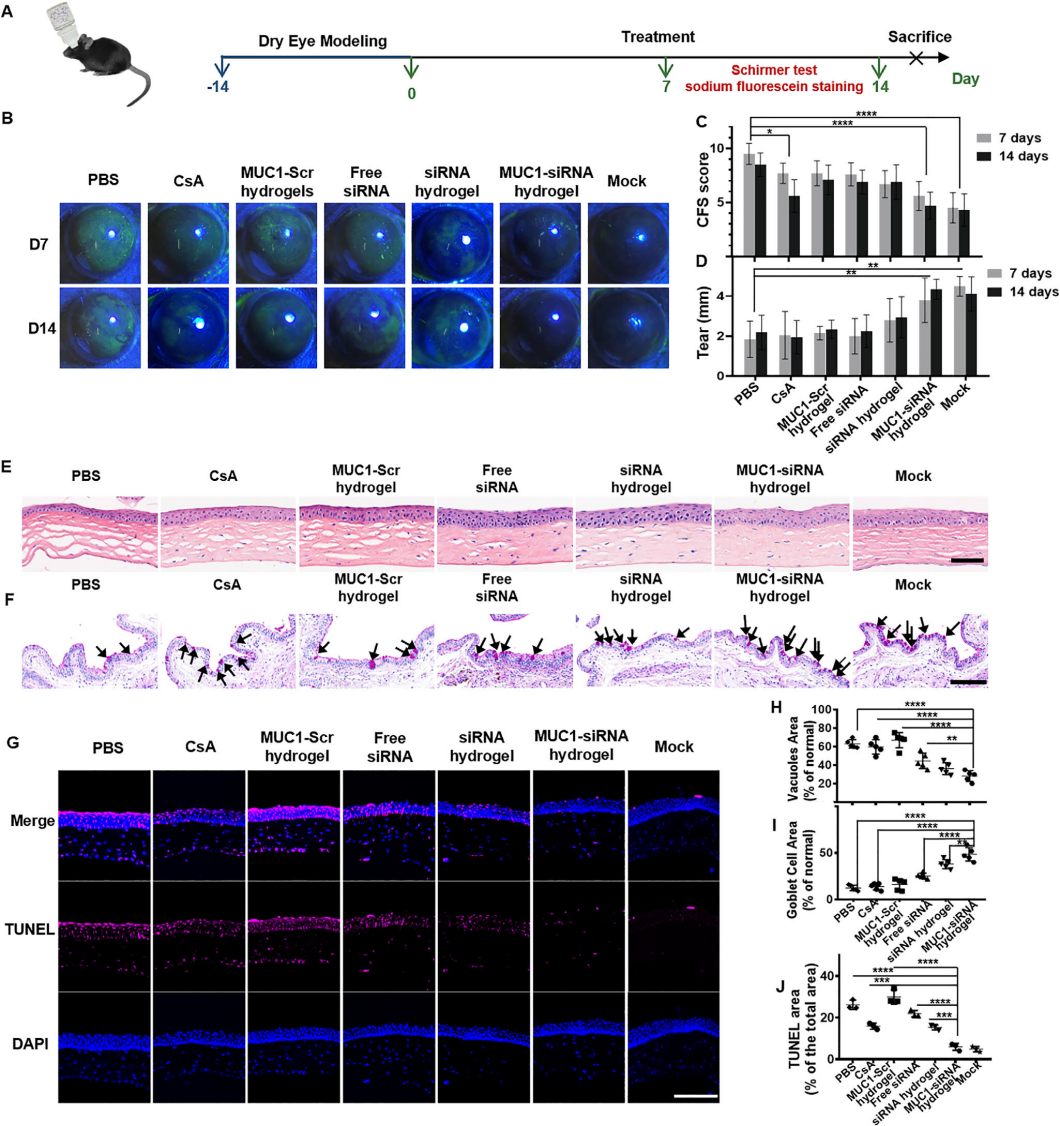
The therapeutic effects of MUC1‐siRNA hydrogel on the DED mouse model. (A) Flow chart of in vivo DED model establishment and the treatment using MUC1‐siRNA hydrogel. (B) Representative CFS images from different groups at Day 7 and Day 14. (C) Corresponding CFS scores based on CFS images and (D) tear secretion determined by the Schirmer test measuring the wetted phenol thread lengths. Data are presented as mean ± SD; *n* = 5. Statistical significance: *p < 0.05, **p < 0.01, and ****p < 0.0001. (E) Representative H&E staining images of the corneas from different groups after the treatments. Scale bars: 100 µm. (F) Representative conjunctival periodic acid‐Schiff (PAS) staining images for eyes treated with different formulas. Blue arrow: goblet cells. Scale bars: 100 µm. (G) Apoptosis of corneal cells in different groups evaluated by TUNEL immunostaining of the corneal cross section (scale bar: 50 µm) after 14‐day treatment. (H) Quantitative analysis of vacuoles area percentage in treated eyes compared with the normal group in the conjunctiva. Data are presented as mean ± SD; *n* = 3. Statistical significance: **p < 0.01, ****p < 0.0001. (I) Quantitative analysis of goblet cell area percentage in treated eyes compared with the normal group in the conjunctiva. Data are presented as mean ± SD; *n* = 3. Statistical significance: ****p < 0.0001. (J) Quantitative analysis of apoptosis of corneal cells in different groups evaluated by TUNEL immunostaining after 14‐day treatment. Data are presented as mean ± SD; *n* = 3. Statistical significance: ***p < 0.001, ****p < 0.0001.

In evaluating the therapeutic effect of MUC1‐siRNA hydrogel, a mouse DED model was established by keeping the mice in a controlled low‐humidity environment for 2 weeks (Figure 5A). Then, the mice received 14 days of continuous topical instillation of 5 µL of PBS, MUC1‐siRNA hydrogel, siRNA hydrogel, MUC1‐Scr hydrogel, free siRNA, and commercial 0.05% CsA eye drops, respectively, in both eyes twice per day (BID). The therapeutic effects were evaluated and recorded on Day 7 and Day 14 during the treatment. Based on corneal fluorescein staining (CFS), which characterizes the damage of corneal epithelium, a positive epithelial defect could be observed after DED model induction. With treatment, the alleviation of CFS could be noticed in the hydrogel groups even on Day 7, especially for the MUC1‐siRNA hydrogel group. On Day 14, the cornea of the MUC1‐siRNA hydrogel group was as clear as that of the mock group, with almost no staining identified. In contrast, all other groups still exhibited spotted staining on the corneal surface (Figure 5B). Meanwhile, statistical CFS scores of the eyes in all experimental groups were collected. As shown in Figure 5C, MUC1‐siRNA hydrogel showed the best efficacy in reducing the CFS scores compared with the control groups, demonstrating its capability to effectively relieve cornea damage. Additionally, the tear secretion test revealed similar results, where the MUC1‐siRNA hydrogel significantly promoted the secreted tear volume after the treatment (Figure 5D). In contrast, 0.05% CsA showed minimal effect in enhancing tear secretion, probably due to its slow onset of action in short treatments. Typically, the DED mice exhibited excessive vacuoles in the corneal stroma, which can be noticed in the hematoxylin and eosin (H&E) staining of the cornea cross‐section. After the MUC1‐siRNA hydrogel treatment, fewer vacuoles were observed with an increased thickness of the epithelial layer compared to those treated with other formulations (Figure 5E,H). On the ocular surface, goblet cells play a key role in soluble mucin secretion, assisting in tear film stabilization. In the DED model, mice exhibited significantly decreased and irregular goblet cells, accounting for mucin deficiency and tear film instability. After the treatment, the goblet cells of the MUC1‐siRNA hydrogel group recovered both in percentage and distribution compared to the control groups (Figure 5F,I). Finally, apoptosis of the cells in both the corneal epithelial and stroma layers was analyzed by transferase‐mediated dUTP nick‐end labeling (TUNEL) staining. As shown in Figure 5G and Figure S10B, the MUC1‐siRNA hydrogel group displayed minimal cell apoptosis, indicating a complete tissue recovery after the treatment. Besides the effects on local ocular tissues, the systemic biosafety of our hydrogel was also evaluated at the end of the in vivo study. Cell cytotoxicity and histological examinations of the major organs of mice after treatment are shown in Figures S9 and S10C, in which no obvious cell and tissue damage was observed for the liver, spleen, and kidneys, confirming the negligible systemic toxicity of our targeting hydrogel. In all, MUC1‐siRNA hydrogel exhibited substantial therapeutic effects on the DED mouse model for corneal epithelial defects, tear secretion, and goblet cell recovery. No obvious toxicity was found on the ocular surface after the treatment.

### 2.6 In Vivo Therapeutic Effects of MUC1‐siRNA Hydrogel on DED Mouse Model

**FIGURE 6:**
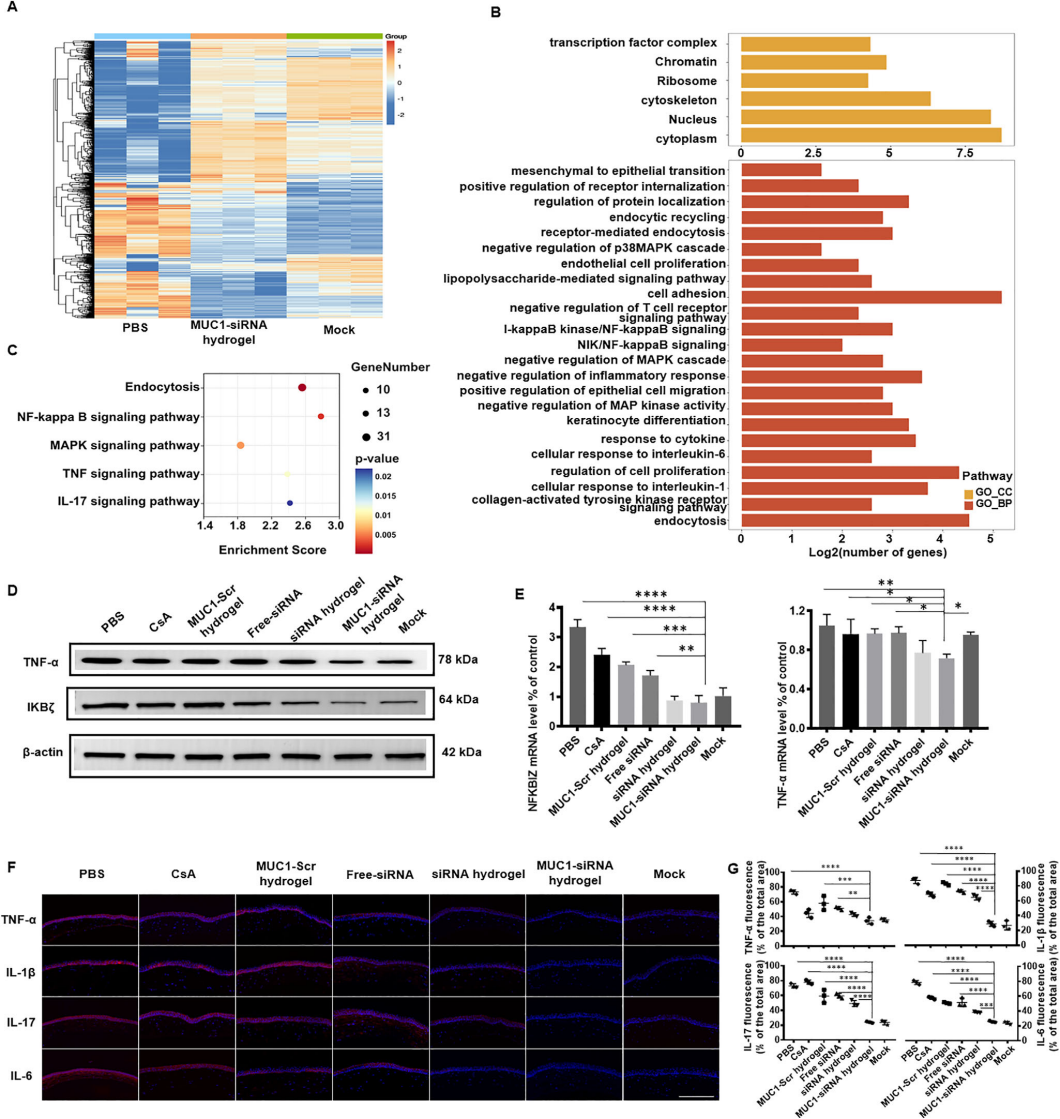
Remolding the corneal gene expression and immune environment of DED through MUC1‐siRNA hydrogel‐induced gene silencing. (A) Heatmap of differently expressed mRNAs (DEmRNAs) in corneas of mice without treatment (mock) and mice treated with PBS and MUC1‐siRNA hydrogel, respectively. (B) Gene ontology enrichment analysis of DEmRNAs in corneas between MUC1‐siRNA hydrogel and PBS‐treated mice. (C) KEGG pathway analysis of DEmRNAs in corneas between MUC1‐siRNA hydrogel and PBS‐treated groups. (D) Western blot analyses to determine the TNF‐α and IKBζ protein expression after treatment with MUC1‐siRNA hydrogel, siRNA hydrogel, MUC1‐Scr hydrogel, free siRNA, and 0.05% CsA eye drops. (E) qRT‐PCR analysis of NFKBIZ and TNF‐α mRNA after treatment with MUC1‐siRNA hydrogel and control formulations. Statistical significance: *p < 0.05, **p < 0.01, ***p < 0.001, ****p < 0.0001. (F) CLSM immunofluorescence imaging of proinflammatory cytokines expressed in the corneas of mice treated with the abovementioned formulations. Scale bar: 150 µm. (G) Quantitative analysis of the proportion of interesting area (%) in the corneas of mice treated with the abovementioned formulations. Data are presented as mean ± SD; *n* = 3. Statistical significance: **p < 0.01, ***p < 0.001, and ****p < 0.0001.

To further elucidate the in‐depth therapeutic action of MUC1‐siRNA hydrogel in treating DED, we conducted RNA‐Seq analysis on corneal tissues from normal mice, DED mice treated with MUC1‐siRNA hydrogel, and those treated with PBS. The heatmap of differentially expressed mRNAs (DEmRNAs) revealed that the expression pattern in the MUC1‐siRNA hydrogel group resembled that of the Mock group but significantly differed from the PBS group, which showed minimal therapeutic effect and maintained the DED condition (Figure 6A). Gene ontology (GO) analysis indicated enrichment of DEmRNAs in cellular components such as the cytoplasm, nucleus, and cytoskeleton, with an emphasis on immune‐related signaling pathways (Figure 6B). Specifically, compared to PBS treatment, the MUC1‐siRNA hydrogel treatment enriched DEmRNAs in endocytic pathways, cell adhesion, and immune‐related pathways. Molecular function GO analysis (Figure S11) revealed involvement of DEmRNAs in activities such as transcription factor activity, DNA binding, RNA binding, and extracellular matrix structural constituent, consistent with therapeutic outcomes. KEGG pathway analysis highlighted alterations in NF‐kappa B, MAPK, TNF‐α, and IL‐17 signaling pathways (Figure 6C), all of which are crucial in immune regulation. Further functional analyses focus on the impact of MUC1‐siRNA hydrogel treatment on genes associated with inflammatory and immune responses (Figure S12). Western blot analysis demonstrated a significant reduction in IKBζ protein and TNF‐α, a proinflammatory cytokine, in the corneas after MUC1‐siRNA hydrogel treatment (Figure 6D), which was corroborated by qRT‐PCR results showing downregulation of NFKBIZ mRNA compared with DED status (Figure 6E), confirming the effective suppression of the NF‐κB signaling cascade. Immunofluorescence staining and the corresponding quantitative analysis revealed minimal presence of proinflammatory cytokines in MUC1‐siRNA hydrogel‐treated corneas, including TNF‐α, IL‐1β, IL‐17, and IL‐6 (Figure 6F,G). Furthermore, evaluation of macrophage polarization was conducted through whole‐mount cornea staining of CD86 and CD206 (Figure S13), which showed a shift towards an anti‐inflammatory M2 phenotype following MUC1‐siRNA hydrogel treatment. Flow cytometry analysis confirmed lower accumulation of Th17 cells in ocular drainage lymph nodes, indicating effective suppression of immune response through NFKBIZ gene silencing (Figure S14). Taken together, efficiently silencing the ocular NFKBIZ gene with MUC1‐siRNA hydrogel suppresses innate immune signaling, reduces proinflammatory cytokine secretion, remodels the DED inflammatory microenvironment, and disrupts the vicious inflammatory cycle of DED, achieving a favorable therapeutic outcome.

## 3 Discussion

The anatomical meaning of the ocular surface refers to the entire mucosal epithelium of the eyeball surface, starting from the gray line between the upper and lower eyelid margins, including the corneal epithelium and the conjunctival epithelium. The most challenging aspect for the controlled ocular delivery of drugs to the eye includes the physiological static barriers, such as the complex layers of the cornea, sclera, and retina, which restrict the drug from permeating into the anterior segments of the eye. Despite multiple available treatment strategies, the effectiveness of conventional ophthalmic formulations is hampered by the presence of physiological barriers, rapid elimination through nasolacrimal drainage, protein binding, and metabolic degradation, which all contribute to reduced ocular residence time and poor bioavailability. In the case of oil‐based CsA formulations (in our control group), those oils used to enhance CsA penetration can be difficult to diffuse to the hydrophilic corneal stroma and rapidly cleared from the eye, resulting in ocular surface irritation and limited bioavailability. Additionally, oxidation of excipients such as arachis oil and castor oil in the formulation can cause significant eye irritation and reduce tolerability and bioavailability [41].

In the present study, we have developed a novel carrier platform for therapeutic siRNA delivery, in which functional siRNA is directly embedded in the self‐assembled nucleic acid hydrogel. Based on the powerful programmable self‐assembly, siRNA against the target gene can be efficiently loaded into the 3D hydrogel network, which may protect the payload from enzymatic degradation. With customized synthesis, aptamers can be conveniently introduced into the siRNA nucleic acid hydrogel, which provides an intriguing strategy to turn the formidable ocular barrier, the cornea and conjunctiva, into a tissue‐specific target for drug delivery. The interactions between the MUC1 aptamer and its target protein allow the MUC1‐siRNA hydrogel to recognize the surface‐associated MUC1 on corneal and conjunctival epithelial cells, which prolongs the residence of the hydrogel at the ocular surface. Furthermore, our self‐assembled nucleic acid hydrogel under normal physiological conditions with adjustable viscosity enhances the retention and bioavailability due to the mucoadhesive properties and is effective for gene delivery from nuclease degradation to controlled‐nanoparticle release in cells. The functional siRNA payloads link to a complementary DNA/RNA hybrid duplex architecture to maintain the capability of RNase H–mediated target mRNA degradation. Upon further studies focused on assessment in vitro and in vivo models, the MUC1‐siRNA hydrogel group demonstrates the most efficiency in inhibiting ocular inflammation through NFKBIZ silencing compared with the other control groups. The downregulation of IκBζ reduces secretion of IL‐1β, TNF‐α, and other chemokines from ocular surface tissues (Figures 3F–I and 6F), which in turn reprograms macrophage polarization from pro‐inflammatory M1 (CD86^+^) to anti‐inflammatory M2 (CD206^+^; Figure S13). This cross‐talk is consistent with our prior findings, where the NFKBIZ knockdown systemically suppressed NF‐κB signaling and reversed the inflammatory microenvironment of the ocular surface in DED [42]. Moreover, the adhesion feature and the binding of aptamers to MUC1 endow our hydrogel with good epithelial tissue targeting and ocular surface retention, which may also facilitate the restoration of tear film stability through mucin‐mediated mechanisms. As demonstrated in Figure 5F–I, treatment significantly recovered the goblet cell density and distribution in the conjunctiva of DED mice. These goblet cells secrete soluble mucins that form a hydrophilic glycocalyx layer on the ocular surface [1], which anchors the tear film's aqueous layer through reducing surface tension via amphiphilic mucin domains, establishing osmotic gradients that regulate aqueous layer distribution, and forming electrostatic networks with tear components to prevent dewetting. Taken together, the restoration of a balanced immune microenvironment and stabilized tear film would disrupt the vicious cycle of inflammation in DED, achieving an ideal therapeutic efficacy.

In all, the self‐assembled targeting hydrogel described here opens a new option for DED treatment, which is absolutely biodegradable, biocompatible, non‐toxic, non‐immunogenic, and easy to use during administration. It may hold the key to solving the aforementioned physiological barrier and low bioavailability challenges in ocular surface delivery. As a general approach, the NFKBIZ siRNA sequence can be easily changed to target other key signaling genes in eye diseases, and the aptamer segments can also be conveniently designed to target other membrane‐associated mucin‐related substrates on the cell surface in the future, which can greatly expand the scope of drug and large‐scale synthesis for engineering.

## 4 Experimental Procedures

### 4.1 Syntheses of MUC1‐siRNA Hydrogel

DNA and RNA strands were obtained from Shanghai Sangon Co., Ltd., China. The concentrations of oligonucleotides were quantified utilizing UV–Vis measurements performed on both the UV‐1800 (Shimadzu Corporation, Japan) and NanoDrop One (Thermo Fisher Scientific, USA). Initially, equimolar quantities of three DNA strands (chains A, B, and C) for each kind of Y‐shaped building block were mixed separately in two tubes with 1× TAE/Mg^2+^ buffer (40 mM Tris, 2 mM EDTA‐Na‐H_2_O, 20 mM acetic acid, and 12.5 mM (CH_3_COO)_2_Mg•4H_2_O, pH = 7.4, adjusted by acetic acid) (Thermo Fisher Scientific, USA), to form the designed Y‐shaped motifs (MUC1 Y‐motifs). The mixtures were heated up to 90°C for 5 min, followed by gradual annealing to room temperature. The NFKBIZ siRNA was prepared by mixing the sense and antisense strands at an equal molar ratio in TAE/Mg^2+^ buffer (diethyl pyrocarbonate water), then heated to 75°C for 10 min and annealed to room temperature. Subsequently, for the fabrication of MUC1‐siRNA hydrogel, the assembly concentration condition was optimized by mixing MUC1 Y‐motifs and NFKBIZ siRNA crosslinkers at a definite molecular ratio of 1:1.5. The different concentrations of MUC1‐siRNA hydrogel were rapidly formed within 1 min. Similarly, non‐target NFKBIZ siRNA hydrogel (siRNA hydrogel), negative control sequence siRNA hydrogel (MUC1‐Scr hydrogel), Cy3‐labeled hydrogel (Cy3‐MUC1‐siRNA hydrogel/Cy3‐siRNA hydrogel), and Cy5.5‐labeled hydrogel (Cy5.5‐MUC1‐siRNA hydrogel/Cy5.5‐siRNA hydrogel) were synthesized using the same aforementioned method (Figure S1). In cell drug delivery and adherence in vivo, our linker uses complete siRNA as mentioned above to ensure rigorous reproducibility and quality control of hydrogel fabrication. In the in vivo treatment experiment, we only need to provide the siRNA linker that can achieve the therapeutic effect, and the remaining linkers are replaced by DNA. This is also due to the targeted effect of the MUC1 aptamer, which brings the benefit of reducing the dosage of the siRNA drug with inter‐batch variability <2.1% (*n* = 5 batches). These samples were analyzed by 20% native PAGE at 1× TAE/Mg^2+^ buffer, running buffer using an FB‐VE10‐1 electrophoresis unit (Fisher Biotech, USA). After electrophoresis, the gels were stained using ethidium bromide (EB; Adamas‐beta, China) and scanned. Following staining, the nucleic acid strands were imaged under a Bio‐Rad imaging system (Bio‐Rad, USA).

### 4.2 Characterizations of MUC1‐siRNA Hydrogel

The morphology of MUC1‐siRNA hydrogel was characterized using field‐emission scanning electron microscopy (SEM; Sirion 200, FEI, USA) after being thoroughly freeze‐dried overnight under vacuum. Rheological tests were performed on a Kinexus ultrat+ rheometer (TA Instruments, USA) equipped with a temperature controller with a 25 mm parallel‐plate configuration. Frequency sweep tests were carried out between 0.1 and 10 rad·s^−1^ with 1% strain at both 25°C and 37°C. The viscosity and injectable property of MUC1‐siRNA hydrogel were verified at 25°C. At a fixed frequency of 1 Hz, cycle tests were performed from low strain 0.1% to high strain 50% and temperature‐ramp tests from 25°C to 60°C. The shear storage modulus (G′) and shear loss modulus (G″) changing with angular frequency and temperature were recorded, respectively. The diameter and morphology of MUC1‐siRNA particles in PBS containing 2.4 mg·mL^−1^ (Beyotime, Shanghai) were characterized by DLS (Zetasizer Nano ZS, Malvern Instruments, UK), TEM (Tecnai G2 Spirit Biotwin, FEI Ltd., USA), and AFM (Nanonavi E‐Sweep, Japan). DLS analysis of MUC1‐siRNA nanogel particles was conducted on an instrument equipped with a 125‐mW laser at 25°C, and the scattering angle was kept at 173°. For TEM, the MUC1‐siRNA nanogel particles were deposited onto copper grids, stabilized for 3 min, excess solution removed, washed twice, stained, and dried by compressed air. AFM imaging involved spotting 5 µL of MUC1‐siRNA nanogel particles onto a mica surface, followed by washing and drying using nitrogen gas.

### 4.3 In Vitro Stability and Release Behavior

By incubating with a tear‐mimic fluid (2.4 mg·mL^−1^ lysozyme in PBS buffer, Sangon Biotech, China), the lysozyme and shear force‐induced degradation of the MUC1‐siRNA hydrogel were evaluated. Briefly, MUC1‐siRNA hydrogel was incubated with above tear‐mimic PBS buffer for 0, 2, 4, 6, 8, and 12 h at 37°C with slow stirring (200 rad·min^−1^) to simulate the ocular surface physiological environment and blinking of the eyes. Their stabilities were analyzed by 15% native PAGE, and the sample was incubated with buffer for 0, 2, 4, 6, 8, and 12 h at 37°C, as mentioned above. And the intracellular MUC1‐siRNA nanogels were incubated with different concentrations of RNase H (0, 1, 2.5, 5, 10, 20, and 25 U·mL^−1^) at 37°C for 1 h to mimic RNase H‐mediated RNA release in cells. The release of functional RNA segments was characterized by 10% denaturing PAGE electrophoresis. After gel electrophoresis, gel images were recorded under a Bio‐Rad imaging system (USA).

### 4.4 Cell Culture

HCEC, HCT‐116, HepG2, mouse fibroblast cells (L929), and human monocyte cells (THP‐1) were cultured at 37°C in a humidified atmosphere containing 5% CO_2_. HCEC, HepG2, and L929 cells were incubated in DMEM culture media containing 10% FBS and antibiotics (50 units·mL^−1^ penicillin and 50 units·mL^−1^ streptomycin). HCT‐116 and THP‐1 cells were cultured in McCoy's 5A medium and RPMI‐1640 medium containing 10% FBS and antibiotics, respectively.

### 4.5 In Vitro Cellular Uptake

HCEC, HCT‐116, HepG2, and L929 cells were seeded in 12‐well plates at 3.0 × 10^5^ cells per well and cultured in DMEM overnight. The Cy3‐labeled siRNA linker hydrogel was added into the wells at a uniform Cy3 concentration (1 µM) and incubated in Opti‐MEM at 37°C for various durations (0, 1.0, 2.0, 4.0, 6.0, and 8.0 h), as well as with different concentrations of Cy3 (0, 1.0, and 2.0 µM) for an 8‐h period. Control groups without target segment hydrogel treatment (Y‐motif siRNA‐Cy3) were also included. Cells were harvested using trypsin and collected for flow cytometry analysis (BD FACSCalibur, USA).

For the CLSM study, HCEC, HCT‐116, HepG2, and L929 cells were seeded at 4 × 10^4^ cells per well in 24‐well culture plates with a clean coverslip put in each well and grown overnight for attachment, followed by incubation with MUC1‐siRNA hydrogel and no target‐segment siRNA hydrogel labeled with Cy3 (equivalent Cy3 concentration: 1 µM) for 8 h. Subsequently, culture medium was removed, and cells were washed three times with PBS and fixed with 4% formaldehyde for 15 min at room temperature. After blocking for 60 min, the cells were immunostained with antibody MUC1 (14161, 1:400; Cell Signaling Technology, Massachusetts, USA) at 4°C overnight. Thereafter, the primary antibody was removed, and the specimens were washed three times and incubated in horseradish peroxidase–conjugated second antibody (7074, 1:3000; Cell Signaling Technology, Massachusetts, USA) solution for 2 h. Following washing and Prolong Antifade Reagent with DAPI (#8961, Cell Signaling Technology, Massachusetts, USA), specimens were observed with a laser scanning confocal microscope (Leica TCS SP8 STED 3X, Germany).

### 4.6 In Vitro Gene Regulatory Function Induced by MUC1‐siRNA Hydrogel

The gene knockdown efficiency was evaluated by the corresponding protein and mRNA expression. Protein expression was evaluated using a Western blot assay. HCECs were seeded in six‐well plates at 5.0 × 10^5^ cells per well and cultured in DMEM overnight. After being pre‐treated with 2 µg·mL^−1^ LPS (Beyotime Biotechnology, China) for 2 h, cells were then co‐cultured with MUC1‐siRNA hydrogel, siRNA hydrogel, MUC1‐Scr hydrogel, and free siRNA (at an equivalent 200 nM siRNA in 2 mL sample‐containing medium. For siRNA‐bearing hydrogel, typically 1 µL of 300 µM siRNA‐bearing hydrogel containing 0.4 nmol siRNA was pre‐mixed in 1 mL culture medium, then another 1 mL culture medium was added), and commercial CsA eye drop (5 µM CsA, by adding 24 µL 0.05% CsA eye drop in 2 mL culture medium) for 8 h. After replacing the culture medium, cells were continuously incubated for another 48 h. The total proteins were extracted from cells using RIPA buffer containing protease inhibitor cocktail. Thereafter, the protein concentrations were determined with bicinchoninic acid protein assay kit (Invitrogen, Carlsbad, CA, USA). Thereafter, 30 µg of proteins were separated by 5%–15% SDS‐PAGE and transferred to 0.22 µm polyvinyl difluoride membranes (Millipore, USA). Subsequently, the membranes were blocked with 5% blotting grade milk and then incubated with primary and secondary antibodies successively. After incubating with antibodies, the protein bands were imaged by electrochemical luminescence (Invitrogen, USA) and quantified by ImageJ software.

For mRNA quantification, a qRT‐PCR assay was performed. First of all, the cells were cultured with drug formulations as described above. Then, fresh growth media were replaced for another 24 h incubation. Total RNA was extracted from cells by TRIzol extraction (Thermo Fisher Scientific Inc.). Subsequently, the corresponding cDNAs were reverse‐transcribed using the AMV reverse transcription system (Promega) following the manufacturer's procedure. The qRT‐PCR experiments were performed on a qRT‐PCR system (Tprofessional thermocycler, Biometra, Germany) using PowerUp SYBR Green Master Mix kit (Thermo Fisher Scientific Inc., USA). The thermal cycling conditions for the PCR program were as follows: 95°C for 3 min, 35 cycles of (95°C for 60 s, 55°C for 50 s, 72°C for 60 s).

THP‐1 cells were spread at 2 × 10^5^ cells·well^−1^ in six‐well plates and stimulated with 100 ng·mL^−1^ phorbol 12‐myristate 13‐acetate (PMA) for 24 h. Cover the Transwell chamber after THP‐1 cells are polarized and adhered. HCECs were spread on the upper chamber of Transwell (Tansoole, China) at 2 × 10^5^ well^−1^ and placed on the polarized THP‐1 cells so that the upper and lower chambers of Transwell were filled with the new cell culture medium with 100 ng·mL^−1^ LPS to simulate the spatial distribution of ocular surface epithelial cells and mononuclear macrophages. Overnight, cells were transfected as mentioned, and the co‐culture cell‐culture medium was collected 24 h later. The cytokine (TNF‐α, IL‐1β, IL‐17, and IL‐6) secretions were quantified according to the manufacturer's instructions of ELISA Kits (Thermo Fisher Scientific, USA).

### 4.7 In Vitro Cytotoxicity Induced by MUC1‐siRNA Hydrogel

The CCK8 assay was applied to evaluate the cytotoxic effects of different groups of hydrogel and samples. HCEC cells were seeded in 96‐well plates at a density of 8 × 10^3^ cells per well and cultured for 24 h. Then the culture medium was replaced by 100 µL fresh medium containing MUC1‐siRNA hydrogel, siRNA hydrogel, MUC1‐Scr hydrogel, and free siRNA eye drops for 48 h. After that, 10 µL of CCK8 (Beyotime Biotechnology, China) reagents were added into each plate and incubated for 2 h. At last, 100 µL solution was transferred to a new 96‐well plate, and the absorbance was measured at 450 nm by BioTek SynergyH4 hybrid reader.

### 4.8 DED Mouse Model and Treatment

All animal experiments have complied with the Association for Research in Vision and Ophthalmology Statement. All animal experiments were performed according to the guidelines of the Fudan University Ethics Committee with the approval number of IRB‐EENT‐150301. Sixty specific‐pathogen‐free C57BL/6 mice (120 eyes) of 6–8 weeks old were selected and housed in an environmentally controlled chamber with adequate rodent chow and water available. These mice were randomized into six groups and exposed to a low‐humidity environment (RH = 18.5% ± 5.1%, AF = 15 L/min^−1^, T = 21–23°C) for 14 days for the dry eye mouse model. Then each group of the DED mice received topical instillation of 5 µL of PBS, MUC1‐siRNA hydrogel, siRNA hydrogel, MUC1‐Scr hydrogel, free siRNA (with an equivalent siRNA dose by administrating a 5 µL sample on each eye), and commercial 0.05% CsA eye drops twice a day in both eyes for 14 days, while kept in a normal environment (RH = 50%–80%, no AF, T = 21°C–23°C). As siRNA can exert significant therapeutic effects at relatively low concentrations, we incorporated only the required amount of siRNA linker in the hydrogels used for treatment in the DED mouse model. The remaining linker for hydrogel self‐assembly was replaced with a DNA linker of the same sequence, resulting in the formation of a 300 µM hydrogel containing 22.5 µM siRNA linker and 277.5 µM DNA linker. For detailed sequences, see Table S1.

### 4.9 Precorneal Retention Evaluation

In vivo imaging with anesthesia and confocal imaging of cryopreserved sections of mice's eyeballs without anesthesia were conducted to evaluate precorneal retention time. For in vivo imaging detection with anesthesia, the C57BL/6 mice were intraperitoneally anesthetized and instilled with 2 µL of Cy5.5‐MUC1‐siRNA hydrogel, Cy5.5‐siRNA hydrogel, and free Cy5.5 (with an equivalent Cy5.5 concentration of 0.3 mM) onto the right eyes. Later, the variation of fluorescence intensity was monitored using the in vivo imaging system (IVIS Spectrum) at distinct time points (0, 0.5, 1, 3, 5, 10, 15, 20, 25, and 30 min) (Ex/Em, 675 nm/720 nm).

For cryopreserved section imaging without anesthesia, the same eye drops of Cy5.5‐MUC1‐siRNA hydrogel, Cy5.5‐siRNA hydrogel, and free Cy5.5 as previously mentioned were installed onto the corneal surfaces of the same mice under consciousness. Then, mice were sacrificed at the precise time points of 2, 10, and 30 min after the administration, soon after which these eyeballs were carefully removed and immediately frozen in optimal cutting temperature (OCT). The 12 µm frozen slices of eyeballs were sectioned and then observed under a fluorescence microscope (10×), and Z‐stack images were acquired through CLSM.

### 4.10 Therapeutic Efficacy Assessment

Basal tear secretion assessment (Schirmer I Test): The Schirmer's *I*‐test was performed using Zone‐Quick Phenol‐Red cotton thread (Yokota, Japan) on Day 7 and Day 14 post‐treatment initiation (21, 28 days after modeling). Following anesthesia intraperitoneally with 1.25% avertin (20 µL·g^−1^), 1 mm of the phenol red cotton end was gently inserted into the lower lateral conjunctival fornix of mice for 30 s. The color of phenol cotton changes from yellow to red, indicating the secretion of tears, and the wetted length (mm) of the strip was read and recorded. The eyelids were closed after examination to avoid overexposure.

Corneal fluorescein sodium staining score: 2 µL of 0.5% sodium fluorescein (w/v) was instilled into the ocular surface, and subsequently, the eyelids were manually closed three to four times before gently removing the excess sodium fluorescein. The fluorescein staining images were captured under a slit lamp with a cobalt blue light, and the corresponding scores were graded by the same experienced oculist. The cornea was divided into five quadrants, and a grade of 0–3 was assigned to each quadrant: 0, absent; 1, punctate staining fewer than 30 spots; 2, punctate staining more than 30 spots, but not diffuse; 3, severe diffuse staining or plaque positive. The sum of all quadrants is regarded as the final score. All eyes in every group were examined and used for analysis.

### 4.11 In Vivo Underlying Mechanisms

The transcriptome sequencing and analysis were conducted by OE Biotech Co., Ltd. (Shanghai, China). Briefly, the corneal tissues' gene libraries were constructed using the VAHTS Universal V6 RNA‐seq Library Prep Kit according to the manufacturer's instructions. Then, the libraries were sequenced on an Illumina Novaseq 6000 platform, and 150 bp paired‐end reads were generated. Differential mRNA expression analysis was performed using DESeq2. *p*‐value < 0.05 and log2FC > 0.585 or log2FC < −0.585 were set as the threshold for significantly differentially expressed genes (DEGs). The bioinformatic analyses of DEGs were performed by the online software Database for Annotation, Visualization and Integrated Discovery (DAVID, https://david.ncifcrf.gov/home.jsp) [43]. The expression of RNA in both groups and the analysis results were visualized using R software.

The corneas of mice were carefully removed for subsequent real‐time qPCR, Western blot, and whole transcriptome RNA sequencing. The total RNA of each cornea was extracted and purified using the RNA extraction kit (EZB‐RN4, EZBioscience, China). The first strand of cDNA was reverse transcribed with the Reverse Transcription kit (A0010CGQ, EZBioscience, China). Quantitative real‐time PCR was examined using the Quanti Nova SYBR Green kit (A0012‐R2, EZBioscience, China) following the manufacturer's instructions. Data were analyzed using the comparative Ct method. GAPDH was used as an internal control.

Western blots were performed as described in a previous study [44]. The following antibodies were used: NFKBIZ (14014‐1‐AP, 1:500; Proteintech, Rosemont, USA), TNF‐α (60291‐1‐Ig, 1:500; Proteintech, Rosemont, USA), and β‐actin (3700, 1:25,000; Cell Signaling Technology, Danvers, MA).

Flow cytometry: Ipsilateral cervical and submandibular draining lymph nodes were collected from each group 14 days after treatment, and single‐cell suspensions were prepared as described [45]. In brief, single‐cell suspensions of lymph nodes were prepared by homogenizing the collected lymph nodes in 70‐µm cell strainers, which were then incubated with an anti‐Fc‐receptor blocking antibody (Clone 93; BioLegend, San Diego, CA) to avoid non‐specific staining. The following monoclonal antibodies were purchased from BioLegend: anti‐mouse CD4 (GK1.5) and IL‐17A (TC11‐18H10.1). For intracellular IL‐17A staining, cells were stimulated in cell culture medium (Thermofisher) with PMA/Ionomycin (MultiSciences, China) and Brefeldin A (BFA)/Monensin (MultiSciences, China) for 5 h following the manufacturer's instructions. After washing, cells were first stained for surface antigens (CD4) and then fixed and permeabilized (IC Fixation Buffer and Permeabilization Buffer; eBioscience, San Diego, CA) before intracellular staining for IL‐17A. Isotype‐matched control antibodies were used in all experiments. Finally, cells were analyzed using a flow cytometer (BD LSRFortessa X‐20), and data were analyzed using FlowJo software X 10.0.7 (Tree Star Inc.).

### 4.12 Immunofluorescence, Tissue Inflammation, and Safety Evaluation

After intraperitoneal anesthesia, the intact eyeballs (including conjunctiva) were collected according to the previous method [46]. The eyeballs were then placed in 4% paraformaldehyde (PFA) for the following cornea H&E staining, conjunctival PAS staining, and immunofluorescence experiments. On the second and third days, eyeballs were placed in 20% and 30% sucrose for gradient dehydration, respectively. OCT embedding was performed subsequently (4583, SAKURA). The frozen sections were dried at room temperature (30 min) and washed with a PBS solution for three times with a 5‐min interval. After a chemical pen creating circles around the tissue, 4% PFA was used to fix the sections for 20 min at room temperature. Then, the sections were immersed in 0.1% Triton X‐100 (in PBS) for 15‐min periods. The terminal deoxynucleotidyl TUNEL mixture (Roche Diagnostics, Indianapolis, IN, USA) was prepared by mixing fluorescein‐labeled dUTP and terminal deoxynucleotidyl transferase (9:1), which was incubated with specimens in a dark box for 60 min at a temperature of 37°C. After blocking, immunofluorescence experiments were performed by staining the sections with primary antibodies (single staining: IkBζ, IL‐6, IL‐17A, IL‐1β, TNF‐α; spread double staining: CD206/CD86), which were incubated overnight at 4°C. After washing with PBS solution for three times with a 5‐min interval again, the slices were blocked at room temperature with 3% bovine serum albumin for 1 h. Finally, the nuclei were stained with DAPI. The images were taken by an SP8 confocal microscope under 40× with an oil‐immersion microscope. In addition, the main tissues (spleen, liver, and kidney) were reserved for H&E staining to evaluate the biosafety of the formulations.

### 4.13 Statistical Analyses

Data were presented as means ± SD. The statistical significance was determined using the analysis of a two‐tailed Student's *t*‐test and one‐way analysis of variance with GraphPad Prism 6.0. Statistical significance was noted as follows: *p < 0.05; **p < 0.01; ***p < 0.001; ****p < 0.0001. Graph analysis was performed using GraphPad Prism 6.0 (GraphPad Software, USA).

## Author Contributions

Conceptualization: C.Z., J.H., Y.J., and X.J.; Methodology: Y.L., Z.Z., X.Z., Y.J., X.J.; Investigation: Y.L., Z.Z., Y.H., F.W., Z.W., K.J.; Funding acquisition: J.H., X.Z., and C.Z.; Writing‐ original draft: Y.L., Z.Z., and C.Z.; Writing – review & editing: All authors.

## Conflicts of Interest

The authors declare no conflicts of interest.

## Supporting information




**Supporting File**: exp270202‐sup‐0001‐SuppMat.docx.

## Data Availability

All data reported in this paper will be shared by the lead contact upon request. Any additional information required to reanalyze the data reported in this paper is available from the lead contact upon request.
