# Supplementary material for: An Ocular Surface‐Targeting Nucleic Acid Hydrogel for Efficient Dry Eye Disease Treatment
Source: Exploration (Beijing). 2026 Jul 15;6(4):20240461. doi: 10.1002/EXP.20240461 (PMC13394018; doi:10.1002/EXP.20240461)
Supplement: Supplementary file 1 [file EXP2-6-20240461-s001.docx]

An Ocular Surface-Targeting Nucleic Acid Hydrogel for Efficient Dry Eye Disease Treatment

Yuhe Liu^1†^, Zimeng Zhai^2†^, Yangyang Huang^1^, Fujun Wang^1^, Zifei Wang^3^, Lijuan Zhu^1^, Ka Jiang^2^, Xujiao Zhou^2^, Xingtao Zhou^2^, Ying Jie^4^, Xiuming Jin^5^, Chuan Zhang^1*^, Jiaxu Hong^2,6,7*†^.

^1^School of Chemistry and Chemical Engineering, Frontiers Science Center for Transformative Molecules, Shanghai Key Laboratory for Molecular Engineering of Chiral Drugs, Shanghai Jiao Tong University, Shanghai, 200240, P. R. China.

^2^Department of Ophthalmology and Vision Science, Shanghai Eye, Ear, Nose and Throat Hospital, State Key Laboratory of Molecular Engineering of Polymers, Fudan University, Shanghai, 200031, P. R. China.

^3^School of Life Sciences, Fudan University, Shanghai, 200438, P. R. China.

^4^Beijing Institute of Ophthalmology, Beijing Tongren Eye Center, Beijing Tongren Hospital, Capital Medical University; Beijing Ophthalmology & Visual Sciences Key Laboratory, Beijing, 100730, China.

^5^Eye Center, the Second Affiliated Hospital of Zhejiang University, School of Medicine, No.1, Xihu Avenue, Hangzhou, 310009, China.

^6^Department of Ophthalmology, Children’s Hospital of Fudan University, Shanghai, 201102, P. R. China.

^7^Department of Ophthalmology, Nan’an Hospital Affiliated to Shanghai University, Quanzhou, 362300, P. R. China.

Author Contributions: † These authors contribute equally.

**This PDF file includes:**

Figures S1 to S14

Tables S1 to S2

**Figure S1.** 20% native polyacrylamide gel electrophoresis to check the formation of MUC1-siRNA hydrogel. Results of hydrogel formation of (A) MUC1-Scr hydrogel; (B) siRNA hydrogel; (C) MUC1-siRNA-Cy3 hydrogel; (D) MUC1-siRNA-Cy5.5 hydrogel.

**Figure S2.** A representative AFM image of MUC1-siRNA hydrogel diluted in lysozyme-containing PBS buffer (2.4 mg·mL^-1^, 100-fold dilution) with slow stirring (200 rad·min^-1^) and incubation under 37 ℃ for 6 hours.

**Figure S3.** Characterizations of hydrogel transformation and stability. (A) The degradation behavior of MUC1-siRNA from hydrogel to nanogels analyzed by 2% agarose gels. (B) Lysozyme-simulated stability of MUC1-siRNA hydrogel with the extension of the time at 37°C by 15% native polyacrylamide gel electrophoresis. The morphology of MUC1-siRNA nanogel determined by TEM at 2 h (C), 4 h (D), 8 h (E) and 12 h (F) incubated in 2.4 mg·mL^-1^ lysozyme -PBS.

**Figure S4.** (A) CLSM images of HCEC cells treated with MUC1-siRNA hydrogel for different incubation time. Blue: DAPI (nucleic stain); Red: FITC (MUC1 membrane-associated mucins); Green: Cy3 (siRNA hydrogel). Scale bars: 100 μm. (B) CLSM images of HCT-116, HepG2, L929 cells treated with 1 μM Cy3-labeled MUC1-siRNA hydrogel. Blue: DAPI (nucleic stain); red: FITC (the MUC1 protein on the membrane surface); green: Cy3. Scale bars: 50 μm.

**Figure S5.** (A) CLSM imaging of HCEC cellular uptake behaviors via inhibition assays. Scale bars: 20 μm. (B) The mean fluorescence intensities of HCEC cellular uptake behaviors via inhibition assays. (Equivalent Cy3 concentration: 1 μM). (C) In vitro HCEC cellular uptake behaviors of MUC1-siRNA hydrogel (siRNA linker was labelled with Cy3 at an equivalent concentration of 1 μM) determined by immunofluorescence (membrane-associated MUC1 was visualized by immunostaining using FITC-labelled MUC1 antibody) and CLSM imaging after 8 h-incubation, and thorough PBS washing at 12 and 24 h. Scale bars: 20 μm. (D) Flow cytometry analysis of HCEC cells treated with MUC1-siRNA hydrogel and incubated for different time after PBS washing at 8 h. (E) The mean fluorescence intensities of HCEC cells treated with MUC1-siRNA hydrogel and incubated for different time after PBS washing at 8 h . (Equivalent Cy3 concentration: 1 μM).

**Figure S6.** Cellular uptake behaviors of MUC1-siRNA hydrogel by HconEpic cells. (A) Flow cytometry analysis of HconEpic cells treated with MUC1-siRNA hydrogel (right) and siRNA hydrogel (left) for different incubation time. (sB) The mean fluorescence intensities of HconEpic cells treated with MUC1-siRNA hydrogel and control sample for different incubation time (with equivalent Cy3 concentration: 1 μM). Data were presented as mean ± SD; n = 3. Multiple t tests statistical significance: **p < 0.01, ****p < 0.0001. (C) In vitro HconEpic cellular uptake behaviors of MUC1-siRNA hydrogel and pristine siRNA hydrogel (siRNA linker was labelled with Cy3 at an equivalent concentration of 1 μM) determined by immunofluorescence (membrane-associated MUC1 was visualized by immunostaining using FITC-labelled MUC1 antibody) and CLSM imaging after 6 h incubation. Scale bars: 20 μm.

**Figure S7.** (A) Western blot analysis of IKBζ and TNF-α expression in HCEC cells after treatment with different concentrations of siRNA hydrogel, MUC1-Scr hydrogel, and siRNA. (B-E) mRNA levels of MMP9, IL-6, IL-8, and p65 expression were determined by quantitative reverse transcription-polymerase chain reaction (qRT-PCR) analyses respectively. Data were presented as mean ± SD; n = 3. Statistical significance: *p < 0.05, **p < 0.01.

**Figure S8.** Supplementary data of gene knockdown and anti-inflammatory effects in vitro. Western blot analysis of IKBζ and MMP9 expression in HCEC cells with co-culture with THP-1 after treatment with varied formulations.

**Figure S9.** Safety and biocompatibility of MUC1-siRNA hydrogel, siRNA hydrogel, MUC1-Scr hydrogel, and free siRNA to HCECs by CCK-8 assay. HCECs were treated for 24 h, followed by CCK-8 assay. Data were presented as mean ± SD; n = 5.

**Figure S10.** Tissue retention, apoptosis and safety analysis in vivo. (A) Fluorescent images of ocular surface frozen sections after administration with MUC1-siRNA-Cy5.5 hydrogel, siRNA-Cy5.5 hydrogel or free Cy5.5 on mice under conscious states for 2, 10, 30 min. The scale bar is 50 μm. (equivalent Cy5.5 concentration: 0.3 mM). (B)Apoptosis evaluation by the terminal transferase-mediated dUTP nick end-labeling (TUNEL) immunostaining on the conjunctival epithelial cells (Scale bar: 25 μm) in the mice eyes after 14 days of various therapies for DED. (C) Histological examination of major organs of the DED-model mice after treatments using different drug formulations (Scale bar: 100 μm).

**Figure S11.** Molecular function gene ontology enrichment analysis of DEmRNAs between MUC1-siRNA hydrogel and PBS treated corneal tissues.

**Figure S12.** Functional analyses from gene ontology (GO) and KEGG events were performed and visualized using the Metascape gene enrichment analysis tool (https://metascape.org).

**Figure S13.** (A) Immunofluorescence staining of the whole-mount corneas after treatment. The staining of M1 macrophages (red, using CD86 as biomarker), M2 macrophages (green, using CD206 as biomarker), and nucleus of corneal cells (blue) were demonstrated for both Mock group and DED mice treated with indicated formulations, scale bar: 1 mm. (B) Quantitative analysis of proportion of staining area (%) in the whole-mount corneas with above-mentioned formulations. Data were presented as mean ± SD; n = 3. Statistical significance: ***p < 0.001, ****p < 0.0001.

**Figure S14.** Supplementary data of anti-inflammatory effects with Th17 cells in vivo. Flow cytometry-based CD4+ Th17 cells analysis of mice lymph nodes.

**Table S1.** Oligonucleotide sequences used in this work.

| DNA or RNA | Sequences (from 5’ to 3’) |
| --- | --- |
| Human-Aptamer chainA | GCAGTTGATCCTTTGGATACCCTGGTTTTTTGGATCCGCATGACATTCGCCGTAAGTTGACCTGTGAA |
| ChainA | TGGATCCGCATGACATTCGCCGTAAGTTGACCTGTGAA |
| ChainB | CTTACGGCGAATGACCGAATCAGCCTTTGACCTGTGAA |
| ChainC | AGGCTGATTCGGTTCATGCGGATCCATTGACCTGTGAA |
| Human-SenseNFKBIZ linker | GCCCGAUUCGUUGUCUGAUUUCACAGGUCAA |
| Human-AntisenseNFKBIZ linker | AUCAGACAACGAAUCGGGCUUCACAGGUCAA |
| Mouse-SenseNFKBIZ linker | GCGUCAAUGUACCAGUAUUUUCACAGGUCAA |
| Mouse-AntisenseNFKBIZ linker | AAUACUGGUACAUUGACGCUUCACAGGUCAA |
| Human-SenseNFKBIZ | GCCCGAUUCGUUGUCUGAU |
| Human-AntisenseNFKBIZ | AUCAGACAACGAAUCGGGC |
| Mouse-SenseNFKBIZ | GCGUCAAUGUACCAGUAUU |
| Mouse-AntisenseNFKBIZ | AAUACUGGUACAUUGACGC |
| Sense scramble linker | UGGUUUACAUGUUGUGUGAUUCACAGGUCAA |
| Antisense scramble linker | UCACACAACAUGUAAACCAUUCACAGGUCAA |
| Mouse-SenseNFKBIZ DNA-linker | GCGTCAATGTACCAGTATTTTCACAGGTCAA |
| Mouse-AntisenseNFKBIZ DNA-linker | AATACTGGTACATTGACGCTTCACAGGTCAA |
| Sense scramble DNA-linker | TGGTTTACATGTTGTGTGATTCACAGGTCAA |
| Antisense scramble DNA-linker | TCACACAACATGTAAACCATTCACAGGTCAA |

The underlined segments were sticky end sequences of the Y-motif and siRNA linkers.

Aptamer sequence was labeled in red.

**Table S2.** Primer sequences used in this work.

| Primer name | Sequences (from 5’ to 3’) |
| --- | --- |
| Human-NFKBIZ forward | CТCAACCTGAGCTACTTCTACG |
| Human-NFKBIZ reverse | AGAGGAGAAGTCGGAGGAG |
| Human-TNFα forward | GGACACCATGAGCACTGAAAGC |
| Human-TNFα reverse | TGCCACGATCAGGAAGGAGAAG’ |
| Human-MMP9 forward | CTGGTCCTGGTGCTCCTGGTG |
| Human-MMP9 reverse | CTGCCTGTCGGTGAGATTGGTTC |
| Human-IL-6 forward | CACTGGTCTTTTGGAGTTTGAG |
| Human-IL-6 reverse | GGACTTTTGTACTCATCTGCAC |
| Human-IL-8 forward | CTCTCTTGGCAGCCTTCCTGATTTC |
| Human-IL-8 reverse | GGGGTGGAAAGGTTTGGAGTATGTC |
| Human-p65 forward | ATGTGGAGATCATTGAGCAGC |
| Human-p65 reverse | CCTGGTCCTGTGTAGCCATT |
| Human-GAPDH forward | GCACCGTCAAGGCTGAGAAC |
| Human-GAPDH reverse | TGGTGAAGACGCCAGTGGA |
| Mouse-NFKBIZ forward | AGAAATACCAACCGTTCCAAGTC |
| Mouse-NFKBIZ reverse | TGCTGCATGTTCTGTGTTTGT |
| Mouse-TNFα forward | GCCACCACGCTCTTCTGTCT |
| Mouse-TNFα reverse | TGAGGGTCTGGGCCATAGAAC |
| Mouse-MMP9 forward | CGCCACCACAGCCAACTATGAC |
| Mouse-MMP9 reverse | CTGCTTGCCCAGGAAGACGAAG |
| Mouse-IL-1β forward | TGGCAACTGTTCCTG |
| Mouse-IL-1β reverse | GGAAGCAGCCCTTCATCTTT |
| Mouse -IL-6 forward | CTTCTTGGGACTGATGCTGGTGAC |
| Mouse -IL-6 reverse | TCTGTTGGGAGTGGTATCCTCTGTG |
| Mouse-IL-17A forward | ATGAGTGCCGACAAACAACG |
| Mouse-IL-17A reverse | GTGACGTGGAACGGTTGAGG |
| Mouse-βactin forward | CACTGTCGAGTCGCGTCC |
| Mouse-βactin reverse | TCATCCATGGCGAACTGGTG |
